# Hypoxia and *TTR* dysregulation in astrocytes from Parkinson’s disease with a specific mitochondrial haplogroup: A single-cell analysis

**DOI:** 10.4103/NRR.NRR-D-25-00107

**Published:** 2025-09-03

**Authors:** Junhao Wang, Wenxuan Du, Xinyi Chen, Hao Wu, Ganqiang Liu

**Affiliations:** 1 Shenzhen Key Laboratory of Systems Medicine in Inflammatory Diseases, School of Medicine, Shenzhen Campus of Sun Yat-sen University, Shenzhen, Guangdong Province, China; 2 Neurobiology Research Center, School of Medicine, Shenzhen Campus of Sun Yat-sen University, Shenzhen, Guangdong Province, China; 3 Guangdong Provincial Key Laboratory of Brain Function and Disease, Guangzhou, Guangdong Province, China

**Keywords:** astrocyte, brain network functional connectivity, cognitive progression, gene regulatory network, haplogroup H, mitochondrial DNA variants, nerve regeneration, Parkinson’s disease, reactive oxygen species, single-nucleus RNA sequencing

## Abstract

Mitochondrial DNA variants have been linked to cognitive progression in Parkinson’s disease; however, the mechanisms by which mitochondrial DNA variants or haplogroups contribute to this process remain unclear. In the present study, we analyzed single-nucleus RNA sequencing data from 241 post-mortem brain samples across five regions to investigate the dysregulatory mechanisms associated with mitochondrial DNA haplogroup H and haplogroups J, T, and U^#^. Our findings revealed significant alterations in the proportions of astrocyte subtypes *CHI3L1* and *GRM3* in the neocortical regions of haplogroup H. Notably, *TTR* was markedly downregulated in the dorsal motor nucleus of the Xth nerve region of patients with haplogroup H. Pathway analysis highlighted abnormal hypoxic and reactive oxygen species environments in astrocytes, whereas protein complex analysis revealed a consistent and significant elevation in ribosomal subunit complexes within the astrocyte subtypes. By constructing weighted and directed transcriptome-wide gene regulatory networks, we identified significant changes in transcription factor SP1 and homeobox protein HOXA5 activity in the astrocyte subtypes of individuals with haplogroup H. Additionally, widespread dysregulation was observed in the transcriptional control of *TTR* by multiple transcription factors. Parkinson’s disease patients with haplogroup H also exhibited increased network functional connectivity in specific brain regions. This data-driven study underscores the potential mechanisms by which mitochondrial DNA haplogroups contribute to cognitive progression in Parkinson’s disease, involving cellular composition changes, differential gene expression, pathway disruption, and gene regulatory networks. Our findings suggest that mitochondrial DNA haplogroup H may drive Parkinson’s disease cognitive progression through aberrant *TTR* expression and a hypoxic environment.

## Graphical abstract

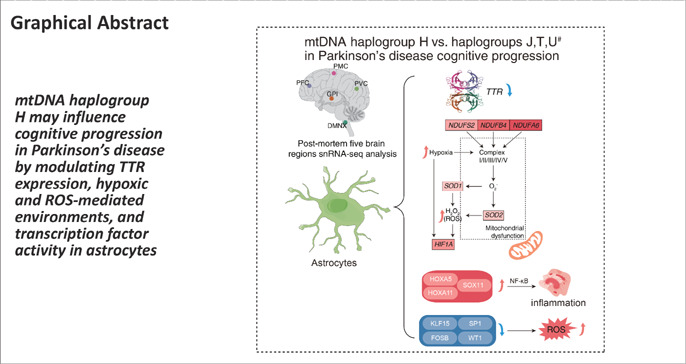

## Introduction

Parkinson’s disease (PD) is the second most prevalent neurodegenerative disorder (Aarsland et al., 2021), and patients exhibit significant clinical heterogeneity in disease progression patterns (Berg et al., 2021). Despite extensive research, a comprehensive understanding of PD progression remains elusive. Genome-wide association studies have identified approximately 100 distinct genes or loci associated with PD susceptibility (Lim et al., 2024); their functions converge on key biological pathways, including synaptic function, lysosomal activity, mitochondrial dysfunction (Coukos and Krainc, 2024), and immune-mediated mechanisms (Tansey et al., 2022). For example, mutations in *LRRK2* (associated with autosomal dominant PD) disrupt autophagy and mitochondrial function (Ye et al., 2023), whereas *CHCHD2* and *CHCHD10* regulate mitochondrial fusion (Ikeda et al., 2022), and *PINK1/PRKN* mediate mitophagy (Narendra and Youle, 2024).

Emerging evidence links mitochondrial DNA (mtDNA) haplogroups to PD susceptibility (Sena-Dos-Santos et al., 2024). Mechanistically, haplogroup H might elevate PD risk through heightened reactive oxygen species (ROS) generation in later life (Hudson et al., 2013), whereas haplogroup J appears to enhance mitochondrial coupling, thereby conferring neuroprotective effects (Gaweda-Walerych et al., 2008). Additionally, *TFAM*—a key regulator of mtDNA metabolism—may modulate ROS production and PD risk independently or via interactions with specific haplogroups, thus contributing to PD pathogenesis (Gaweda-Walerych et al., 2010). The mtDNA haplogroup background within cells significantly influences their epigenetic profiles, such as global methylation, as well as genes related to acetylation and methylation (Atilano et al., 2015). A recent genetic survival analysis revealed that European PD patients carrying mitochondrial haplogroups J, T, or U^#^ have a 41% lower risk of cognitive decline than those with haplogroup H (Liu et al., 2023). Despite these insights, the precise mechanisms by which mtDNA haplogroups or variants influence PD progression remain unclear.

During α-synuclein-mediated neurodegeneration, astrocytes exhibit distinct region-specific activation patterns that differentially modulate neuroinflammatory responses (Basurco et al., 2023; Edison, 2024). The preservation and enhancement of mitochondrial function in astrocytes may mitigate PD progression through the dual suppression of oxidative stress and chronic neuroinflammation (Wang et al., 2023). Notably, the dorsal motor nucleus of the Xth nerve (DMNX) is a critical region for pathological α-synuclein deposition and serves as a key relay center for α-synuclein spread from central to peripheral tissues (Pinto-Costa et al., 2023) or from the body to the brain (Horsager et al., 2020; Yang and Zhang, 2024). Oxidative stress in the DMNX may accelerate α-synuclein oligomer formation and propagation (Musgrove et al., 2019). The influence of mtDNA haplogroups on mitochondrial oxidative stress has also been described (Antonyova et al., 2020). Thus, understanding the molecular mechanisms underlying these changes across different brain regions, cell types, and mtDNA haplogroups will be critical for deciphering the mechanisms of cognitive decline in PD.

Herein, we applied single-cell analysis and various analytical approaches to explore brain region- and cell type-specific effects of mitochondrial haplogroups in PD patients. Specifically, we examined five distinct anatomical regions from PD patients with mitochondrial haplogroup H and haplogroups J, T, and U^#^ to investigate the potential molecular mechanisms by which mtDNA haplogroups influence PD progression.

## Methods

### Haplogroup classification

Whole-genome sequencing data were obtained from the Accelerating Medicines Partnership PD (AMP-PD) project (https://app.terra.bio/#workspaces/amp-pd-public/AMP-PD-In-Terra) (N M et al., 2024), comprising 100 post-mortem brain donors collected from four brain banks: Mount Sinai Brain Bank, University of Miami Brain Endowment Bank, Harvard Brain Tissue Resource Center, and Udall Center of Excellence for PD Research. There was a similar distribution of sex and age for each brain bank. The cohort comprised 75 PD donors stratified using Braak PD staging, which tracks the spread of Lewy body pathology, and 25 neuropathologically confirmed controls. Genomic DNA was extracted from the primary visual cortex (PVC) for whole-genome sequencing. Mitochondrial haplogroup calling was then performed using the HaploGrep3 (v.3.2.1) (https://github.com/genepi/haplogrep3) (Schönherr et al., 2023) command line tool, using VCF files as the input and the PhyloTree17—Forensic Update (rCRS Human mtDNA) Version 1.2 (phylotree-fu-rcrs@1.2) (https://github.com/genepi/phylotree-rcrs-17) as the reference tree. Based on the output from HaploGrep3, the 100 individuals were classified into 16 haplogroups. Additionally, large fragment deletions of mtDNA were assessed for every individual using eKLIPse (https://github.com/dooguypapua/eKLIPse) (Goudenège et al., 2019). Building on evidence that mitochondrial haplogroups J, T, and U^#^ (including all U haplogroups U1–U7 but excluding haplotype K) confer reduced risk for cognitive decline compared with haplogroup H (Liu et al., 2023), we analyzed a targeted cohort of 51 PD patients stratified across these haplogroups, to evaluate their differential effects on PD-associated cognitive progression. *P*-values for continuous and categorical variables were determined using two-sample *t*-test and Pearson’s chi-squared test with simulated *P*-values (**Table 1**). Additionally, all 51 PD cases in the cohort were systematically screened for common PD risk gene variants, including *LRRK2* (rs33949390, R1628P; rs34637584, G2019S; rs34778348, G2385R), *SNCA* (rs104893875, E46K; rs104893878, A30P), and *GBA* (rs421016); no cases were detected with these risk single nucleotide polymorphisms.

**Table 1:** Characteristics of post-mortem PD patients between haplogroup H and haplogroups J, T, and U^#^

	H (*n* = 30)	JTU (*n* = 21)	*P*-value
Age, yr	79.13±6.30	79.24±5.61	0.95^a^
Sex, *n* (%)			0.40^b^
Male	9 (30.0)	9 (42.9)	
Female	21 (70.0)	12 (57.1)	
Braak stages, *n* (%)			0.23^b^
II	0 (0)	2 (9.5)	
III	9 (30.0)	4 (19.0)	
IV	9 (30.0)	3 (14.3)	
V	9 (30.0)	9 (30.0)	
VI	3 (10.0)	3 (14.3)	
Brain banks, *n* (%)			0.43^b^
HA	9 (30.0)	3 (14.3)	
MS	3 (10.0)	5 (23.8)	
UD	10 (33.3)	8 (38.1)	
UM	8 (26.7)	5 (23.8)	
Family PD history, *n* (%)			0.78^b^
Yes	1 (3.3)	2 (9.5)	
No	9 (30.0)	7 (33.3)	
Unknown	20 (66.7)	12 (57.1)	

^a^*P* values were calculated using two-sample *t*-test, whereas ^b^*P* values were derived from the Pearson’s chi-square test with simulated *P*-values. HA: Harvard Brain Tissue Resource Center; MS: Mount Sinai Brain Bank; PD: Parkinson’s disease; UD: Udall Center of Excellence for Parkinson’s Disease Research; UM: University of Miami Brain Endowment Bank. Data are expressed as number (percentage) and mean ± SD, as appropriate.

Data used in the preparation of this article were obtained on August 23, 2021 from the Parkinson’s Progression Markers Initiative (PPMI) database (www.ppmi-info.org/access-data-specimens/download-data, RRID: SCR_006431). For up-to-date information on the study, visit www.ppmi-info.org. The mitochondrial haplogroups of PD patients in the PPMI were obtained from our previous study (Liu et al., 2023). Samples belonging to haplogroups H, J, T, and U^#^ were extracted for subsequent T1-weighted magnetic resonance imaging (MRI) and resting-state functional MRI (rs-fMRI) analysis (**Additional Tables 1** and **2**).

**Additional Table 1:** Baseline demographic and clinical characteristics of patients with PD in the T1 analysis

	H (*n* =117)	J, T, U^#^(*n* =136)	*P* value^&^
Age, yr	62.93 ± 9.77	61.26 ± 10.13	0.2238
Sex, M/F, *n* (%)	77(65.81)/40(34.19)	78(57.35)/58(42.65)	0.1696
Years of education	15.03 ±3.37	15.45 ±3.06	0.4532
PD duration*, yr	2.68 ±2.38	2.75 ±2.18	0.7213
MDS-UPDRS II	6.41 ±4.92	6.04± 4.15	0.8883
MDS-UPDRS III	21.04 ± 8.27	20.31 ±8.99	0.2716
MoCA	27.11 ±2.05	27.45 ± 2.33	0.0872
GDS	2.36 ±2.07	2.33 ±2.65	0.2403
H&Y stage	2 (1-2)	2 (1-3)	0.5455

*PD duration is calculated by subtracting the symptom onset age from the T1 scan age. ^&^*P* value is obtained from the Mann-Whitney *U* test. Data are presented as number (percentage), mean ± standard deviation (continuous data) or median and range (ordinal data). F: Female; GDS: Geriatric Depression Scale; H&Y: Hoehn and Yahr; M: male; MDS-UPDRS: Movement Disorder Society Unified Parkinson’s Disease Rating Scale; MoCA: Montreal Cognitive Assessment; PD: Parkinson’s disease.

**Additional Table 2:** Baseline demographic and clinical characteristics of patients with PD in the rs-fMRI analysis

	H (*n* = 26)	J, T, U^#^ (*n* = 42)	*P* value^&^
Age, yr	59.80 ±11.02	59.77 ± 10.82	0.992
Sex, M/F, *n* (%)	17(65.38)/9(34.62)	31(73.81)/11(26.19)	0.4699
Years of education	14.92 ±2.87	15.52 ±2.94	0.383
PD duration*, yr	3.92 ± 3.88	3.86 ±2.15	0.522
MDS-UPDRS II	5.28 ± 4.01	5.10 ±3.92	0.8156
MDS-UPDRS III	18.32 ± 8.42	18.59 ± 8.86	0.9578
MoCA	27.36 ±2.10	27.90 ±2.15	0.1813
GDS	1.76 ± 1.39	2.67 ±2.86	0.9622
H&Y stage	1 (1-2)	2 (1-2)	0.5773

*PD duration is calculated by subtracting the symptom onset age from the rs-fMRI scan age. ^&^*P* value is obtained from the Mann-Whitney *U* test. Data are presented as number (percentage), mean ± standard deviation (continuous data) or median and range (ordinal data). F: Female; GDS: Geriatric Depression Scale; H&Y: Hoehn and Yahr; M: male; MDS-UPDRS: Movement Disorder Society Unified Parkinson’s Disease Rating Scale; MoCA: Montreal Cognitive Assessment; PD: Parkinson’s disease; rs-fMRI: resting-state functional magnetic resonance imaging.

### Single-nucleus RNA sequencing data preprocessing and quality control

Five post-mortem brain regions were selected for the single-nucleus RNA sequencing (snRNA-seq) data, with the chosen regions representing the progression of PD from pre-motor signs to end stages. The five regions were as follows: the DMNX and globus pallidus interna, which are usually affected in early PD, and the primary motor cortex (PMC), dorsolateral prefrontal cortex (PFC), and PVC, which are involved in late-stage PD.

The snRNA-seq data processing, clustering, and cell type annotation were performed using SCANPY (V1.10.4) (https://github.com/scverse/scanpy) (Wolf et al., 2018) through a four-step quality control (QC) pipeline. The first QC stage implemented stringent cell-level filtering criteria: (1) unique molecular identifier counts (1500–110,000), (2) gene counts (1100–12,500 genes), and (3) mitochondrial gene percentage (< 2%). Ambient RNA contamination was assessed by monitoring non-coding RNA signatures, including ribosomal RNA, small non-coding RNA, pseudogenes, and the long non-coding RNA *MALAT1*. Potential doublets were identified and removed using Scrublet (v.0.2.3) (https://github.com/swolock/scrublet) with default parameters (Wolock et al., 2019). The second QC stage filtered lowly expressed genes (detection rate < 0.05% across nuclei). The third QC step excluded cell types with < 50 cells to ensure robust downstream analysis. The final QC step removed mismatched cells based on a comparison of cell annotation results from the reference and our annotation results. Finally, a dataset was obtained containing 241 samples from five brain regions and 1.15 million cells from 51 PD patients. Gene counts were normalized to the total counts per cell, multiplied by 10,000, and then log-transformed. Highly variable genes were identified using specific filtering parameters (min_mean = 0.0125, max_mean = 3, min_disp = 0.5), and these genes were used to compute low-dimensional embeddings via Uniform Manifold Approximation and Projection (default parameters using 50 principal components and 15 nearest neighbors). Clustering was performed using the Leiden algorithm (resolution = 0.04, 10 iterations), resulting in preliminary cell clusters.

### Single-cell RNA sequencing cluster marker analysis and cell-type annotation

Cell-type annotation was conducted using CellTypist (V1.6.1) (https://github.com/Teichlab/celltypist) (Dominguez Conde et al., 2022), a logistic regression classifier optimized with the stochastic gradient descent algorithm. First, we trained the model using an snRNA-seq dataset from the PFC of the brain, annotated as part of the ROSMAP project (http://compbio.mit.edu/ad_aging_brain/) (Mathys et al., 2023). The same QC procedures applied to our dataset were also implemented for the ROSMAP dataset before training using the celltypist.train function. Subsequently, the trained model was used to predict cell types and subtypes across the five brain regions studied, based on the celltypist.annotate function with the parameter “majority_voting = True” to provide consistent labels. The predicted cell types and subtypes were further confirmed by comparing them with known marker genes specific to each cell type and subtype, thus ensuring the accuracy of the annotation. To clearly distinguish subtypes within each major cell type, marker gene analysis was performed to identify uniquely expressed genes within each cluster. Subtype names were then assigned according to the most prominent and biologically meaningful marker gene identified (e.g., astrocyte subtypes *CHI3L1*, *DPP10*, and *GRM3*) (**Additional Figure 1G–K**). We compared the annotated cell types and subtypes with previously published annotation results and removed cells with inconsistent annotations.

### Single-cell RNA sequencing transcriptomic analysis

We calculated compositional differences by modeling the numbers of specific cell types or subtypes (region-specific per individual) relative to the total cell counts using a quasi-binomial regression model. We assessed the significance of regression contrasts using the emmeans package (V 1.10.1) in R (https://github.com/cran/emmeans) (Searle et al., 2012), and *P*-values were adjusted using the p.adjust function with the false discovery rate (FDR) method.

Differential expression analysis was conducted using the R package Libra (V1.0.0) (https://github.com/neurorestore/Libra) (Squair et al., 2021) with a pseudo-bulk strategy for enhanced statistical power. Cell types with > 50 cells in each group were retained for analysis. Raw counts were aggregated across individual samples within each cluster to generate pseudo-bulk profiles, thereby enabling the application of robust bulk RNA-seq framework with edgeR. Using this approach, single-cell measurements for each study participant and cell type were summarized by summing raw counts. Differential expression analysis was then conducted using the edgeR method implemented in Libra. Confounding factors, including age at death, sex, sourced brain banks, Braak stages, and RNA integrity number, were adjusted, and *P*-values were corrected using the FDR method.

We performed gene set enrichment analysis using the R package irGSEA (V1.1.3) (https://github.com/chuiqin/irGSEA), which uses four distinct methods (AUCell (Aibar et al., 2017), Ucell (Andreatta and Carmona, 2021), ssgsea (Yi et al., 2020), and singscore (Foroutan et al., 2018) to calculate gene set scores. To comprehensively evaluate our findings, we used the robust rank aggregation algorithm (Kolde et al., 2012) from the RobustRankAggreg package (V1.2.1) (https://github.com/raivokolde/RobustRankAggreg/) to integrate the results of the differential analysis. This approach allowed us to identify gene sets that exhibited significant enrichment across multiple gene set enrichment analysis methods.

We also analyzed gene regulatory networks using the R package SCORPION (V1.0.2) (https://github.com/kuijjerlab/SCORPION) (Osorio et al., 2024), which uses coarse-graining of snRNA-seq data to reduce sparsity and enhance the detection of underlying correlation structures within the gene regulatory network. Linear regression was used to analyze the links between transcription factors and their target genes. Transcription factor activity, defined as the sum of all β coefficients (outdegree) for a given transcription factor and its target genes, was compared using the *t*.test function from the Rfast (V2.1.0) package (https://github.com/RfastOfficial/Rfast). *P*-values from multiple comparisons were adjusted using the FDR method.

### Participants and magnetic resonance imaging acquisition from the Parkinson’s Progression Markers Initiative database

Mitochondrial haplogroup stratification identified 253 PD patients from the PPMI (H, *n* = 117; J, T, U^#^, *n* = 136) with available baseline T1-weighted MRI acquired on 3.0-T Siemens platforms (Trio-A-Tim/Verio/Prisma; 1 mm isotropic voxels). Functional analysis was performed on 68 PD patients (H, *n* = 26; J, T, U^#^, *n* = 42) with rs-fMRI data collected using an interleaved acquisition protocol (repetition time = 2400 ms, echo time = 25 ms, 3.3 mm^3^ voxels, 40 slices, and 210 volumes over 8 minutes). All neuroimaging protocols followed standardized PPMI acquisition parameters (https://www.ppmi-info.org/study-design/research-documents-and-sops). Demographic characteristics (i.e., age, education, age at onset, and disease duration) and clinical scales, including motor severity (Movement Disorder Society Unified Parkinson’s Disease Rating Scale parts II and III), cognitive status (Montreal Cognitive Assessment), depressive symptoms (Geriatric Depression Scale), and disease staging (Hoehn and Yahr stage) were extracted from a December 2024 PPMI data freeze. *P*-values were determined using the Mann–Whitney *U* test. Imaging data encompassed baseline T1-weighted MRI scans, temporally matched T1-weighted MRI scans for each participant, and first rs-fMRI scans (0–2 years from baseline).

### T1-weighted magnetic resonance imaging analysis

The voxel-based morphometry technique was implemented in the Computational Anatomy Toolbox12 (CAT12) (http://www.neuro.uni-jena.de/cat/, version 8.1) within Statistical Parametric Mapping (SPM12; https://www.fil.ion.ucl.ac.uk/spm/software/spm12/, version 7771) using MATLAB (version 2020a) (https://ww2.mathworks.cn/help/install/ug/install-products-with-internet-connection.html). Preprocessed T1-weighted MRI scans with the default set of parameters, including diffeomorphic anatomic registration through exponentiated lie algebra algorithm normalization and modulation for nonlinear components (Farokhian et al., 2017). Using the voxel-based morphometry technique, we generated density images of gray matter (GM), white matter (WM), and cerebrospinal fluid (CSF) for each T1-weighted MRI scan, and then smoothed these images with an 8-mm kernel. We also computed the total volumes of GM, WM, and CSF, and the total intracranial volume for each subject. The quality of MRI processing and segmentation was assessed using the “Check homogeneity function” in CAT12 as part of a quality assurance check. Haplogroup-specific GM volume comparisons (haplogroup H vs. J, T, U^#^) were performed using the two-sample *t*-test, corrected for age at scan, years of education, and total intracranial volume. A *P* < 0.05 FDR correction threshold was applied for the voxel-based morphometry analyses.

### Resting-state functional magnetic resonance imaging analysis

The analysis of rs-fMRI data was performed using CONN (Whitfield-Gabrieli and Nieto-Castanon, 2012) (RRID: SCR_009550) release 22.v2407. Functional and anatomical data underwent standardized preprocessing through an integrated neuroimaging pipeline (Nieto-Castanon, 2020), including susceptibility distortion correction during realignment, slice timing correction, outlier detection, direct segmentation, Montreal Neurological Institute space normalization, and smoothing. Functional data were realigned using the SPM “Realign & Unwarp” procedure. Temporal misalignments between different slices of functional data were corrected following the SPM slice-timing correction procedure and potential outlier scans were identified using Artifact Detection Tools (www.nitrc.org/projects/artifact_detect/). Functional and anatomical data were normalized into standard Montreal Neurological Institute space, segmented into GM, WM, and CSF tissue classes, and resampled to 2-mm isotropic voxels. The functional data were smoothed using spatial convolution with a Gaussian kernel of 6-mm full-width half maximum. In addition, functional data were denoised using a standard denoising pipeline (Power et al., 2014) followed by bandpass frequency filtering of the blood-oxygen-level-dependent (BOLD) time series between 0.008 and 0.09 Hz. A component-based noise correction method (CompCor) (Behzadi et al., 2007) was used, and noise components within the WM and CSF were estimated by computing the average BOLD signal as well as the largest principal components orthogonal to the BOLD average, motion parameters, and outlier scans within the eroded segmentation masks of each subject.

Network-based seed-to-voxel analysis using 17 brain networks defined using the Yeo-17 network template (Yeo et al., 2011): Visual Central, Visual Peripheral, Somatomotor A (SomMotA), Somatomotor B, Dorsal Attention A, Dorsal Attention B, Salience/Ventral Attention A, Salience/Ventral Attention B, Limbic A, Limbic B, Control A (ContA), Control B, Control C, Temporal Parietal, Default A, Default B, and Default C. Our network consisted of multiple distinct regions of interest. For the seed-to-voxel connectivity analysis, we obtained the averaged time series of all regions of interest within the network seeds. Connectivity was measured as the correlation between the average time series of all voxels within a network region of interest and the time series of each voxel in the brain. Functional connectivity strength was represented by Fisher-transformed bivariate correlation coefficients derived from a weighted general linear model. The general linear model was adjusted for age at scan, years of education, disease duration, average motion, and average GM probability. Statistical inferences were conducted at the cluster level, and were defined as contiguous voxel groups. Cluster-level inferences were based on parametric statistics using Gaussian random field theory (Worsley et al., 1996). Results were thresholded using a combination of a cluster-forming *P* < 0.001 voxel-level threshold and an FDR *P* < 0.05 cluster-size threshold.

### Ethics statement

Data from multiple cohorts were for used for secondary research in the current study. The Institutional Review Board of the School of Medicine, Sun Yat-sen University (approval No. IRB[2024]39) approved the present analyses; written informed consent by the patients was waived.

## Results

### Multiregional single-cell atlas of mitochondrial haplogroups in Parkinson’s disease

**Figure 1:**
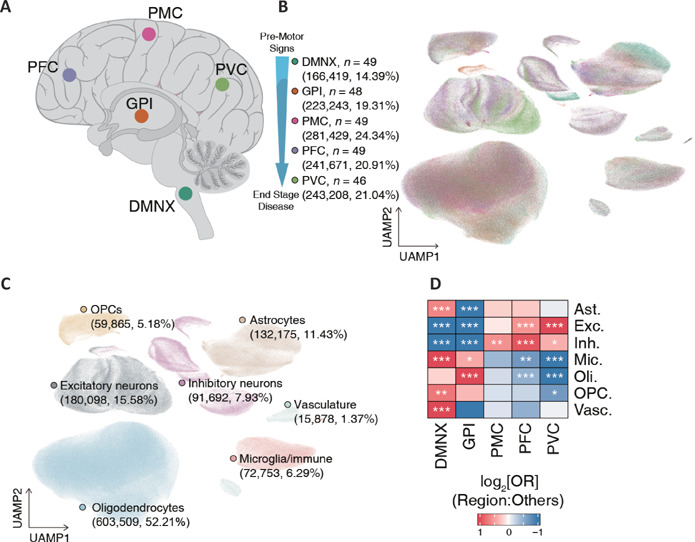
Single-nucleus RNA sequencing (snRNA-seq) analysis of Parkinson’s disease (PD) brain samples. (A) Summary of snRNA-seq profiles, covering 241 PD post-mortem brain samples across five brain regions from 51 patients in the Accelerating Medicines Partnership PD (AMP-PD) Project. (B) Joint Uniform Manifold Approximation and Projection (UMAP) of 1.15 million cells across five brain regions (dorsal motor nucleus of the Xth nerve (DMNX), *n* = 49; globus pallidus interna (GPI), *n* = 48; primary motor cortex (PMC), *n* = 49; dorsolateral prefrontal cortex (DLPFC), *n* = 49; primary visual cortex (PVC), *n* = 46). (C) UMAPs of seven major cell classes, including astrocytes (Ast.), excitatory neurons (Exc.), inhibitory neurons (Inh.), microglia/immune cells (Mic.), oligodendrocytes (Oli.), oligodendrocyte precursor cells (OPC.), and vascular and epithelial cells (Vasc.). (D) Relative enrichment of major cell types across regions by quasi-binomial regression. False discovery rate (FDR)-corrected *P* values are indicated by asterisks; *P < 0.05, **P < 0.01, ***P < 0.001.

To explore the cellular diversity of haplogroups in the human brain and investigate how mtDNA variants contribute to cognitive progression in PD, we performed snRNA-seq on samples from 51 PD patients across five brain regions, obtained from the AMP-PD project (*n* = 241; **Figure 1A**). Based on the whole-genome sequencing data obtained from the PVC region at the time of death, the 51 patients were classified into haplogroup H and haplogroups J, T, and U^#^. No significant differences in age, sex, Braak stages, sourced brain banks, or family PD history were observed between haplogroup H and haplogroups J, T, and U^#^ (**Table 1**), thus minimizing the potential effects of clinical variables on study outcomes. Using Leiden clustering, marker gene analysis, and comparisons with previously published datasets (Materials and Methods), we identified and annotated 54 high-resolution subtypes spanning seven major categories: astrocytes, excitatory neurons, inhibitory neurons, microglia/immune cells, oligodendrocytes, and vascular/epithelial cells, comprising 1 155 970 cells (**Figure 1B** and **C** and **Additional Figure 1**). We next examined the distribution of major cell types across the five brain regions. In neocortical regions, including the PMC, PFC, and PVC, the proportion of excitatory and inhibitory neurons was significantly increased (**Additional Figure 1C**). The DMNX region exhibited pronounced changes in cell proportions; astrocytes, microglia, oligodendrocyte precursor cells, and vascular cells were significantly enriched compared with other brain regions (**Figure 1D**). By contrast, the proportion of neurons (both excitatory and inhibitory) was lower in the DMNX (**Additional Figure 1D**).

### Differences in cell type composition and gene expression between haplogroups

**Figure 2:**
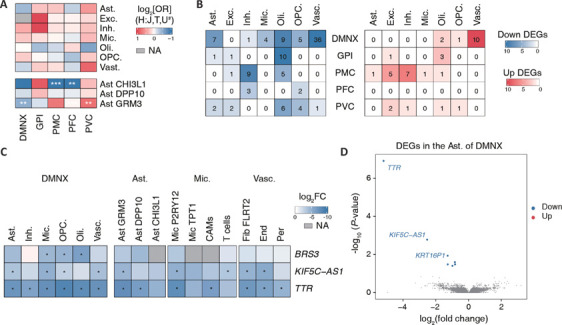
Gene expression changes associated with haplogroups. (A) Relative enrichment of major cell types between haplogroup H and haplogroups J, T, and U^#^ assessed using quasi-binomial regression. FDR-corrected *P* values are indicated: **P < 0.01, ***P < 0.001. (B) The number of significantly differentially expressed genes (adjusted *P* value < 0.05) across seven major cell types. (C) Genes significantly downregulated in specific cell types of the DMNX brain region. *FDR-corrected *P* < 0.05. (D) Volcano plot showing differentially expressed genes in DMNX astrocyte cells between haplogroup H and haplogroups J, T, and U^#^. Genes with significantly higher expression in haplogroup H are shown in red, while those with lower expression are shown in blue. Ast.: astrocytes; DEG: differentially expressed gene; DMNX: dorsal motor nucleus of the Xth nerve; Exc.: excitatory neurons; FDR: false discovery rate; GPI: globus pallidus interna; Inh.: inhibitory neurons; Mic.: microglia/immune cells; Oli.: oligodendrocytes; OPC.: oligodendrocyte precursor cells; PD: Parkinson’s disease; PFC: prefrontal cortex; PMC: primary motor cortex; PVC: primary visual cortex; Vasc. : vascular and epithelial cells.

We next investigated whether brain cell types exhibit compositional differences between haplogroup H and haplogroups J, T, and U^#^. An analysis of the seven major cell types revealed no significant differences in cell type composition between the two groups (**Figure 2A**). Notably, however, astrocyte subtypes exhibited heightened sensitivity to mtDNA variations (**Figure 2A** and **Additional Figure 2**). In the DMNX region, all three astrocyte subtypes showed a trend toward reduced proportions in patients belonging to mtDNA haplogroup H compared with haplogroups J, T, and U^#^. Notably, the proportion of astrocyte *CHI3L1* cells was significantly lower in the PMC and PFC regions of haplogroup H, whereas astrocyte *GRM3* cells showed a significant increase in the PVC region (**Figure 2A**).

To further examine the effects of mtDNA variations on brain cell types, we analyzed differential gene expression across the seven major cell types. After adjusting for covariates, only a small number of genes were significantly associated with haplogroups in any cell type (**Figure 2B**). The DMNX region exhibited a higher enrichment of differentially expressed genes compared with other brain regions, including three genes (*BRS3*, *KIF5C-AS1*, and *TTR*) that were significantly downregulated in more than two cell types within the haplogroup H samples (**Figure 2C**). *BRS3*, which encodes a G-protein-coupled receptor involved in thermoregulation and energy balance (Pinol et al., 2021), exhibited widespread downregulation across all brain regions and cell types in haplogroup H (**Additional Figure 3A**). *KIF5C-AS1* displayed a similar expression pattern (**Additional Figure 3B**). By contrast, *TTR* showed region-specific expression changes (**Additional Figure 3C**). For example, *TTR* exhibited significantly stronger downregulation in DMNX astrocytes from haplogroup H compared with haplogroups J, T, and U^#^ (**Figure 2D**) but showed an upregulation trend across different cell types in the PVC region (**Additional Figure 3C**). Notably, *TTR* expression was consistently low across all brain regions in haplogroup H (**Additional Figure 4B–F**), whereas in astrocytes of haplogroups J, T, and U^#^, its expression gradually declined from the DMNX to the PVC region (**Additional Figure 4A**).

### Effects of cellular pathways and protein complexes in haplogroup H

**Figure 3:**
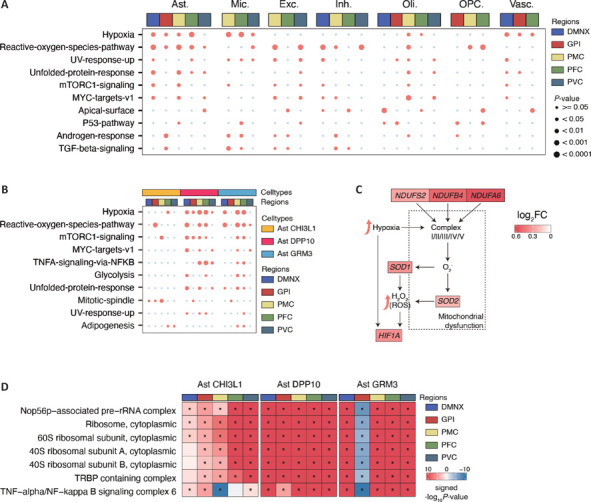
Pathway and protein complex enrichment within haplogroups. (A) Bubble plot showing hallmark pathways significantly upregulated in the seven major cell types of haplogroup H compared with haplogroups J, T, and U^#^. Pathway analysis was performed using robust rank aggregation (RRA), and *P* values were calculated via a two-sided Wilcoxon rank-sum test. (B) Bubble plot illustrating hallmark pathways significantly upregulated in three astrocyte subtypes of haplogroup H compared with haplogroups J, T, and U^#^. (C) Hypoxia and reactive oxygen species (ROS) regulation by HIF-1α. Boxes are color-coded based on the log_2_ fold change (log_2_FC) in gene expression in the PMC Astrocyte DPP10. (D) Heatmap showing the association scores between the expression of seven protein complexes (rows) and haplogroups across three astrocyte subtypes (columns). The signed log_10_ false discovery rate (FDR)-adjusted *P*-value was used, with the sign reflecting the direction (positive or negative) of the association. *FDR-corrected *P* < 0.05. Ast.: Astrocytes; DMNX: dorsal motor nucleus of the Xth nerve; Exc.: excitatory neurons; GPI: globus pallidus interna; Inh.: inhibitory neurons; Mic.: microglia/immune cells; Oli.: oligodendrocytes; OPC.: oligodendrocyte precursor cells; PFC: prefrontal cortex; PMC: primary motor cortex; PVC: primary visual cortex; Vasc.: vascular and epithelial cells.

To further explore the effects of mtDNA variations on cellular pathways, we performed a gene set enrichment analysis on 50 hallmark pathways. This analysis revealed an upregulation of gene set scores across various cell types in haplogroup H patients compared with the haplogroups J, T, and U^#^ group (**Figure 3A**). Among the major cell types, astrocytes exhibited greater enrichment for genes involved in the hypoxia and ROS pathways. Within the three astrocyte subtypes, the *DPP10* and *GRM3* subtypes were similarly enriched for hypoxia and ROS pathways (**Figure 3B**). Additionally, pathways regulating hypoxia, such as mammalian target of rapamycin complex and nuclear factor (NF)-κB, were significantly enriched in astrocyte subtypes across multiple brain regions in haplogroup H patients relative to the haplogroups J, T, and U^#^ group. *HIF1A*, which is a gene that activates pro-inflammatory factors and regulates cellular autophagy, showed increased expression in the astrocyte subtypes of haplogroup H patients compared with the haplogroups J, T, and U^#^ group. Moreover, multiple genes involved in the mitochondrial respiratory chain and ROS production, including *SOD1* and *SOD2*, were upregulated in haplogroup H compared with the haplogroups J, T, and U^#^ group (**Figure 3C**).

To assess whether mtDNA variations affect protein complexes beyond the mitochondrial respiratory chain, we computed module scores for 3684 human protein complexes from CORUM (Giurgiu et al., 2019) and tested their association with haplogroups. Compared with haplogroups J, T, and U^#^, various ribosomal subunit complexes were consistently and significantly elevated across astrocyte subtypes from multiple brain regions in haplogroup H (**Figure 3D**). Overall, single-cell resolution analysis revealed mitochondrial/ribosomal dysregulation in the astrocyte subtypes of haplogroup H patients.

### Alterations in transcription factor activity between haplogroups

**Figure 4:**
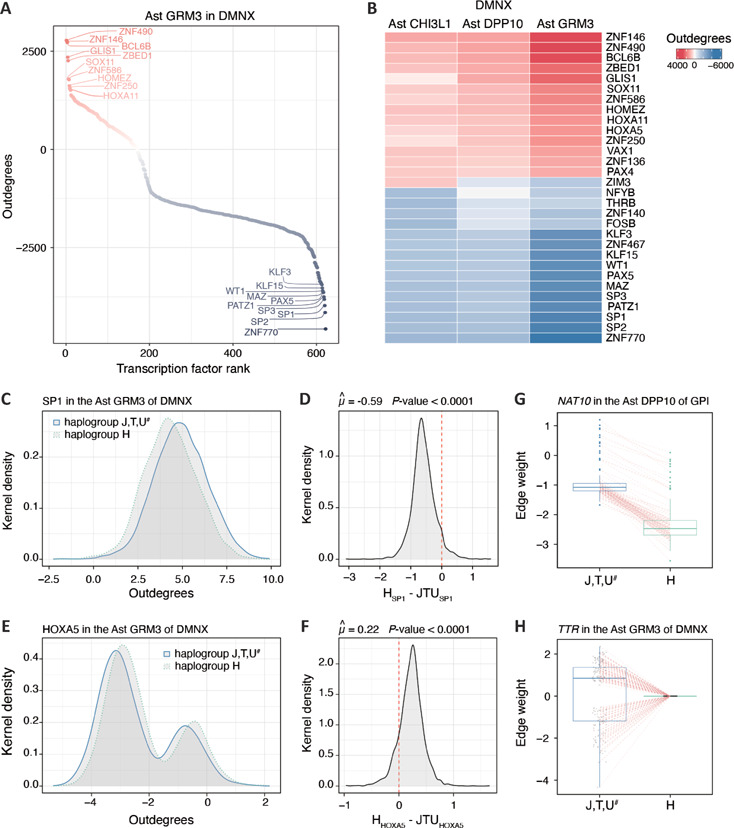
Transcription factor activity between haplogroups. (A) Ranked list of transcription factors in the dorsal motor nucleus of the Xth nerve (DMNX) astrocyte (Ast) GRM3 subtype based on the transcription factor activity in the gene regulatory networks. (B) Heatmap showing the most significantly altered transcription factor activities in three astrocyte subtypes within the DMNX brain region. (C) Comparison of edge weight distributions for the SP1 transcription factor in the Ast GRM3 subtype of the DMNX region between haplogroup H and haplogroups J, T, and U^#^. (D) Distribution of paired weight differences for SP1 edges, with 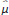 and *P*-values calculated using a one-sample two-sided *t*-test. (E) Comparison of edge weight distributions for the HOXA5 transcription factor in the Ast GRM3 subtype of the DMNX region between haplogroup H and haplogroups J, T, and U^#^. (F) Distribution of paired weight differences for HOXA5 edges, with 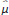 and *P*-values calculated using a one-sample two-sided *t*-test. (G) Boxplot showing significantly reduced interactions between transcription factors and the target gene *NAT10* in the GPI Ast DPP10 subtype between haplogroup H and haplogroups J, T, and U^#^. (H) Boxplot showing significantly imbalanced interactions between transcription factors and the target gene *TTR* in the DMNX Ast GRM3 subtype between haplogroup H and haplogroups J, T, and U^#^.

To further investigate the impact of mtDNA variations on transcription factors, we used SCORPION to generate gene regulatory networks for the astrocyte subtypes that had at least 30 cells across 241 samples. We conducted 503 transcriptome-wide gene regulatory networks, encompassing the regulatory effects of 622 transcription factors on 6980 target genes, amounting to 4 341 560 regulatory links. To assess the influence of haplogroups on transcription factor activity within the astrocyte subtypes, we calculated the overall transcription factor association as the sum of all β coefficients (outdegree) for the links of each transcription factor with its target genes (**Figure 4A** and **Additional Figure 5A** and **B**). Our analysis revealed consistent patterns of transcription factor activity changes across the three astrocyte subtypes in the DMNX region (**Figure 4B**), with the most pronounced alterations observed in the astrocyte *GRM3* subtype. Specifically, in PD patients carrying haplogroup H compared with the haplogroups J, T, and U^#^ group, the activity of transcription factor SP1—whose inhibition increases apoptosis and mitochondrial ROS levels (Gong et al., 2023)—was significantly reduced (**Figure 4C** and **D**). Conversely, the activity of homeobox protein HOXA5, which upregulates the phosphatidylinositol 3-kinase/protein kinase B signaling cascade (Kim et al., 2021), was markedly increased in haplogroup H patients (**Figure 4E** and **F**). Forkhead box protein O family transcription factors, which are critical regulators of antioxidant defense and mitophagy, and nuclear respiratory factor 1 showed attenuated activity in the astrocyte subtypes of haplogroup H relative to the haplogroups J, T, and U^#^ group (**Additional Figure 6A–J**). Conversely, the activity of hypoxia-inducible factor 1-alpha (HIF1A) showed a modest increase (**Additional Figure 6K** and **L**). Additionally, multiple other transcription factors, including KLF15, KLF3, SP3, and FOSB, showed significantly reduced regulatory activities in haplogroup H astrocytes (**Figure 4B**). We further examined the effects of 622 transcription factors on their target gene expression across different haplogroups and identified 334 significant changes in astrocyte subtypes (**Additional Table 3** and **Additional Figure 5C**). Notably, the regulatory strength of 23 transcription factors on the *NAT10* target gene was significantly reduced in the astrocyte *DPP10* subtype of haplogroup H patients compared with the haplogroups J, T, and U^#^ group (**Figure 4G**). Moreover, 159 transcription factors exhibited imbalanced regulatory strength on the *TTR* target gene within the astrocyte *GRM3* subtype of haplogroup H patients relative to the haplogroups J, T, and U^#^ group (**Figure 4H**), suggesting a distinct transcriptional dysregulation.

### Altered functional connectivity of brain networks in Parkinson’s disease patients with haplogroup H

To explore differences in brain structure and network functional connectivity between PD patients with mtDNA haplogroup H and haplogroups J, T, and U^#^, we analyzed T1-weighted MRI and rs-fMRI imaging data from the PPMI cohort. The T1 imaging analysis included 253 patients with PD (**Additional Table 1**), and revealed no differences in GM volume between the two patient groups.

**Figure 5:**
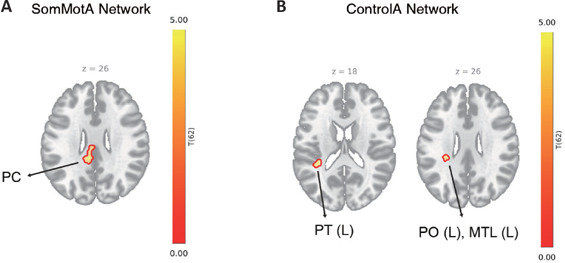
Brain functional connectivity maps comparing Parkinson’s disease patients within haplogroup H to those within haplogroups J, T, and U^#^. Significant increases in brain functional connectivity were observed in haplogroup H (*n* = 26) compared with haplogroups J, T, and U^#^ (*n* = 42) when the (A) SomMotA network and (B) ControlA network were used as seeds. Z indicates the position of the slice (z-coordinate). ContA: Control A network; L: left hemisphere; MTL: medial temporal lobe; PC: posterior cingulate; PO: parietal operculum cortex; PT: planum temporale; SomMotA: somatomotor A network.

For the rs-fMRI analysis, comprising 68 PD patients (**Additional Table 2**), we observed enhanced functional connectivity of the SomMotA and ContA networks within some brain regions in PD patients with mitochondrial haplogroup H (**Table 2**). Specifically, increased functional connectivity was detected between the SomMotA network and the posterior cingulate cortex (FDR *P* = 0.0266; **Figure 5A**). Within the ContA network, haplogroup H patients exhibited enhanced functional connectivity in three brain regions compared with the haplogroups J, T, and U^#^ group: the left parietal operculum cortex, left planum temporale, and left medial temporal lobe (FDR *P* = 0.0155, **Figure 5B**).

**Table 2:** Clusters with significant differences between Parkinson’s disease patients with haplogroup H and those with haplogroups J, T, and U^#^

Seeds	Clusters	Cluster coordinates (x, y, z)	Cluster size	Brian regions of voxel distribution	Cluster FDR *P*-value
SomMotA	1	–16	126	PC	0.0266
ContA	1	–54	103	PO (L), PT (L), MTL (L)	0.0155

The cluster contains multiple voxels. The cluster coordinates are the center coordinates of the cluster, (x, y, z), given at the Montreal Neurological Institute. The cluster size is the number of voxels. ContA: Control A network; L: left hemisphere; MTL: medial temporal lobe; PC: posterior cingulate; PO: parietal operculum cortex; PT: planum temporale; R: right hemisphere; SomMotA: somatomotor A network. The threshold of *P* < 0.05 was corrected for FDR at the cluster level.

## Discussion

A large-scale cohort study indicated that mtDNA haplogroups should be considered a risk factor for cognitive progression in PD (Liu et al., 2023). Here, we conducted a comprehensive analysis of 1.15 million cells across five brain regions of 51 post-mortem PD patients carrying diverse mitochondrial haplogroups: H, J, T, and U^#^. Using robust analytical approaches, we elucidated the potential molecular mechanisms underlying the faster cognitive progression of PD patients with mtDNA haplogroup H compared with those with haplogroups J, T, and U^#^.

In the present study, single-cell analysis identified the DMNX as a region that is particularly susceptible to haplogroup H-associated molecular perturbations; it demonstrated a concerted downregulation of *TTR* across different major cell types and exhibited the highest degree of *TTR* downregulation. *TTR* encodes a thyroid hormone transporter with neuroprotective functions in Aβ clearance (Chu et al., 2023), and regulates axon growth and organelle transport in sensory neurons (Eira et al., 2021). Clinically, low *TTR* plasma levels are correlated with elevated heart failure and mortality risk (Shetty et al., 2024). A previous study reported accelerated cognitive impairment in a young murine model upon *TTR* deficiency (Sousa et al., 2007), and *in vitro* evidence indicates that *TTR* reduces the formation of harmful Aβ aggregates through proteolysis in cultured hippocampal neurons, thereby protecting them from neurotoxicity (Silva et al., 2017). Our findings highlight the potential of *TTR* as a critical mediator between mitochondrial haplogroup H-associated vulnerabilities and PD cognitive progression. A more in-depth study of *TTR* function in neurons and glial cells among PD patients with different haplogroups, potentially using advanced technologies (such as an induced pluripotent stem cell organoid model), will push the boundaries of our understanding of the roles of mtDNA variants in cognitive progression in PD.

Pathway enrichment analysis revealed significant upregulation of hypoxia and ROS pathways in multiple cell types of haplogroup H patients, with astrocytes and their subtypes exhibiting the highest sensitivity. It has been suggested that hypoxia-related pathway upregulation can affect the PD-related protein α-synuclein, thus potentially accelerating Lewy pathology and mitochondrial dysfunction (Burtscher et al., 2021). Similarly, increased ROS levels can cause DNA double-strand breaks, apoptosis, and reduced cellular antioxidant defenses (Dash et al., 2024), further elevating overall ROS levels (Xiao et al., 2022). Hypoxia also upregulates HIF-1α (Gao et al., 2023), and HIF-1α can regulate autophagy and apoptosis (Zhang et al., 2019). Together, these results suggest that rapid cognitive progression in PD patients with haplogroup H may be associated with more damage in the brain microenvironment as a result of hypoxia and ROS. The observed upregulation of *HIF1A* and *SOD2* in haplogroup H cells, especially within the DMNX region, may underlie the accelerated cognitive decline noted in PD. A previous study reported that cholinergic neurons in the DMNX exhibit heightened vulnerability to α-synuclein pathology under oxidative stress conditions (characterized by ROS accumulation) (Musgrove et al., 2019). ROS not only exacerbates oxidative modifications and α-synuclein oligomerization but also promotes its intercellular propagation from the DMNX to more rostral brain regions, thereby promoting the development and progression of PD (Musgrove et al., 2019). Collectively, these molecular alterations underscore the important role of mitochondrial dysfunction and oxidative stress in PD pathogenesis, particularly in patients with specific mitochondrial haplogroups (such as haplogroup H).

Transcription factors can regulate gene expression by binding to sequence-specific promoter regions of target genes, and their roles in PD are increasingly being recognized. In our analysis of transcriptional regulation, we observed consistent regulatory patterns across astrocyte subtypes, and identified significant alterations in the DMNX brain region. This finding highlights both the sensitivity of DMNX astrocytes and their potential contribution to mitochondrial haplogroup-dependent PD cognitive progression. Notably, Krueppel-like factor 15 (KLF15) activity exhibited significant downregulation, which is consistent with previous reports of heightened NF-κB signaling activation in cancer (Chen et al., 2025) and cardiac oxidative stress (Li et al., 2021). Mechanistically, the coordinated suppression of KLF15 and SP1 impairs acetyl-coenzyme A synthetase 2, which is a key enzyme for mitochondrial oxidative metabolism via the tricarboxylic acid cycle (Yamamoto et al., 2004). Other transcription factors, including SP1 (Priya Dharshini et al., 2020), protein FOSB (Zhao et al., 2021), and prothrombin (Ahuja et al., 2024), are also implicated in ROS-mediated inflammation. Furthermore, HOXA5 transcription factor may exert growth-inhibitory effects by activating p53 expression and inducing apoptosis in breast tumors (Raman et al., 2000). Interestingly, our analysis revealed the nearly universal dysregulation of transcriptional control over *TTR* in the astrocyte *GRM3* subtype within the DMNX region of PD patients with haplogroup H. This dysregulation likely contributes to the significantly reduced *TTR* expression observed across multiple cell types in the DMNX region of haplogroup H patients.

An exploratory neuroimaging analysis revealed that the SomMotA and ContA networks in mitochondrial haplogroup H exhibited enhanced functional connectivity with certain brain regions compared with haplogroups J, T, and U^#^. The SomMotA network, which is critical for motor control, body perception, and coordination, encompasses regions such as the primary motor cortex, premotor cortex, and supplementary motor area (Zhang et al., 2021). In haplogroup H patients, the SomMotA network demonstrated increased functional connectivity with the posterior cingulate cortex, which is a key component of the default network (Raichle et al., 2001). Notably, previous studies have reported that the default and somatomotor networks typically exhibit anti-correlation in healthy individuals (Mohan et al., 2016; Dosenbach et al., 2025); increased functional connectivity between these networks may therefore indicate dysfunction, potentially leading to overlapping roles or synchronized activation. Additionally, a negative correlation between the default and somatomotor networks has also been observed to shift toward a positive correlation with aging (Rodriguez-Sabate et al., 2019), suggesting that aging-related changes might be more pronounced in individuals within mitochondrial haplogroup H.

The present study has several limitations. First, the small number of cells and the absence of precise marker genes for excitatory and inhibitory neurons in the DMNX and globus pallidus interna regions limited our ability to perform robust subgroup analyses. Thus, subtype analyses of excitatory and inhibitory neurons were conducted exclusively in the cortical regions (PMC, PFC, and PVC). Second, our neuroimaging analysis was based on a small sample size, highlighting the need for validation in independent cohorts. Third, the current study focused primarily on patients of European ancestry, meaning that the effects of mtDNA haplogroups in other populations remain unclear. Fourth, we examined only transcriptomic changes associated with haplogroups in post-mortem brains. Future research that integrates multi-omics approaches, such as epigenomics and proteomics, might provide greater insights into the roles of mtDNA haplogroups in cognitive progression in PD.

In conclusion, many hurdles remain for obtaining a comprehensive understanding of the functional mechanisms of mtDNA variants in the progression of PD. On the basis of differential gene expression, gene set enrichment, and transcription factor activity analyses, our findings suggest that the DMNX region in patients with haplogroup H exhibits greater mechanism-specific sensitivity than other brain regions. This data-driven study highlights the potential role of mtDNA haplogroups in PD progression through an integrated analysis of multi-region single-nucleus transcriptomic and neuroimaging data. Our findings indicate that PD patients with haplogroup H exhibit an abnormal hypoxic and ROS environment, accompanied by significantly reduced *TTR* expression in astrocytes; together, this may contribute to the faster progression of patients with haplogroup H compared with the haplogroups J, T, and U^#^ group. Collectively, the results of our analysis offer novel insights into disease pathogenesis and provide potential therapeutic targets for PD patients with specific mitochondrial haplogroups.

## Additional files:

***Additional Figure 1:*** *Overview of the post-mortem brain sample and major cell type annotations.*

Additional Figure 1 Overview of the post-mortem brain sample and major cell type annotations.
(A) Joint Uniform Manifold Approximation and Projection (UMAP) representation of 1.1 million cells across seven major cell types, colored and labeled by 54 high-resolution subtypes. (B) UMAP across four haplogroups (H, *n* = 146; J, *n* = 24; T, *n* = 32; U^#^, *n* = 39). (C) The representation of cell types across brain regions, with stacked bar plots showing the proportion of cells contributed by each major cell type across the five brain regions. (D) The representation of brain regions across cell types, with stacked bar plots showing the proportion of cells contributed by each brain region for the seven major cell types. (E) Representation of individuals across cell types, with stacked bar plots showing the proportion of cells contributed by each participant across the seven major cell types. (F) Dot plot presenting the scaled average expression of known cell type marker genes in seven major cell types. (G) Dot plot presenting the scaled average expression of known marker genes in immune cell subtypes. (H) Dot plot presenting the scaled average expression of known marker genes in vascular and epithelial cell subtypes. (I) Dot plot presenting the scaled average expression of known marker genes in astrocyte subtypes. (J) Dot plot presenting the scaled average expression of known marker genes in inhibitory neuron subtypes. (K) Dot plot presenting the scaled average expression of known marker genes in excitatory neuron subtypes.

***Additional Figure 2:*** *Cellular composition analysis.*

Additional Figure 2 Cellular composition analysis.
Relative enrichment of subtypes between haplogroup H and haplogroups J, T, and U^#^, as assessed using quasi-binomial regression, with blue indicating a decrease in the cell proportion in haplogroup H, red indicating an increase, and NA indicating insufficient cells to obtain results.

***Additional Figure 3:*** *Expression profiles of significant genes across brain regions and cell types differs between haplogroup H and haplogroups J, T, and U*^*#*^*.*

Additional Figure 3 Expression profiles of significant genes across brain regions and cell types differs between haplogroup H and haplogroups J, T, and U^#^.
The expression distribution of the genes *BRS3* (A), *KIF5C-AS1* (B), and *TTR* (C) across brain regions and cell types differs between haplogroup H and haplogroups J, T, and U^#^, with blue indicating downregulation in haplogroup H and red indicating upregulation.

***Additional Figure 4:*** *Expression distribution of the TTR gene.*

Additional Figure 4 EExpression distribution of the *TTR* gene.
(A) The expression distribution of the *TTR* gene in astrocyte cells across brain regions. Violin plots showing the expression distribution of the *TTR* gene in seven major cell types across the DMNX (B), globus pallidus interna (GPI) (C), primary motor cortex (PMC) (D), prefrontal cortex (PFC) (E), and primary visual cortex (PVC) (F) brain regions. The cell types with more than 500 cells are shown.

***Additional Figure 5:*** *Distribution of transcription factor activity in astrocyte (Ast) subtypes.*

Additional Figure 5 Distribution of transcription factor activity in astrocyte (Ast) subtypes.
(A) Ranked list of transcription factors in the dorsal motor nucleus of the Xth nerve (DMNX) Ast chitinase-3-like-1 (CHI3L1) subtype based on the transcription factor activity in the gene regulatory networks. (B) Ranked list of transcription factors in the DMNX Ast DPP10 subtype based on the transcription factor activity in the gene regulatory networks. (C) The number of significantly altered links between transcription factors and target genes across three astrocyte subtypes across brain regions.

***Additional Figure 6:*** *Differential edge weight distributions of transcription factors between haplogroup H and haplogroups J, T, and U*^*#*^ *in the Ast GRM3 subtype of DMNX.*

Additional Figure 6 Differential edge weight distributions of transcription factors between haplogroup H and haplogroups J, T, and U^#^ in the Ast GRM3 subtype of DMNX.
Comparison of edge weight distributions for the (A) FOXO1, (C) FOXO4, (E) FOXO3, (G) FOXO6, (I) NRF1, and (K) HIF1A transcription factors in the astrocyte (Ast) GRM3 subtype of the DMNX region between haplogroup H and haplogroups J, T, and U^#^. Distribution of paired weight differences for (B) FOXO1, (D) FOXO4, (F) FOXO3, (H) FOXO6, (J) NRF1, and (L) HIF1A edges, with ^ū^ and *P* values calculated using one-sample two-sided *t*-test.

***Additional Table 1:*** *Baseline demographic and clinical characteristics of patients with PD in the T1 analysis.*

***Additional Table 2:*** *Baseline demographic and clinical characteristics of patients with PD in the rs-fMRI analysis.*

***Additional Table 3:*** *Significant links between transcription factors affecting their target gene expression.*

Additional Table 3 Significant links of transcription factors affecting their target gene expression

## Data Availability

*All relevant data are within the paper and its Additional files.*

## References

[R1] Aarsland D, Batzu L, Halliday GM, Geurtsen GJ, Ballard C, Ray Chaudhuri K, Weintraub D (2021). Parkinson disease-associated cognitive impairment. Nat Rev Dis Primers.

[R2] Ahuja N, Gupta S, Arora R, Bhagyaraj E, Tiwari D, Kumar S, Gupta P (2024). Nr1h4 and Thrb ameliorate ER stress and provide protection in the MPTP mouse model of Parkinson’s. Life Sci Alliance.

[R3] Aibar S, Gonzalez-Blas CB, Moerman T, Huynh-Thu VA, Imrichova H, Hulselmans G, Rambow F, Marine JC, Geurts P, Aerts J, van den Oord J, Atak ZK, Wouters J, Aerts S (2017). SCENIC: single-cell regulatory network inference and clustering. Nat Methods.

[R4] Andreatta M, Carmona SJ (2021). UCell: Robust and scalable single-cell gene signature scoring. Comput Struct Biotechnol J.

[R5] Antonyova V, Kejik Z, Brogyanyi T, Kaplanek R, Pajkova M, Talianova V, Hromadka R, Masarik M, Sykora D, Miksatkova L, Martasek P, Jakubek M (2020). Role of mtDNA disturbances in the pathogenesis of Alzheimer’s and Parkinson’s disease. DNA Repair (Amst).

[R6] Atilano SR, Malik D, Chwa M, Caceres-Del-Carpio J, Nesburn AB, Boyer DS, Kuppermann BD, Jazwinski SM, Miceli MV, Wallace DC, Udar N, Kenney MC (2015). Mitochondrial DNA variants can mediate methylation status of inflammation, angiogenesis and signaling genes. Hum Mol Genet.

[R7] Basurco L, Abellanas MA, Ayerra L, Conde E, Vinueza-Gavilanes R, Luquin E, Vales A, Vilas A, Martin-Uriz PS, Tamayo I, Alonso MM, Hernaez M, Gonzalez-Aseguinolaza G, Clavero P, Mengual E, Arrasate M, Hervas-Stubbs S, Aymerich MS (2023). Microglia and astrocyte activation is region-dependent in the alpha-synuclein mouse model of Parkinson’s disease. Glia.

[R8] Behzadi Y, Restom K, Liau J, Liu TT (2007). A component based noise correction method (CompCor) for BOLD and perfusion based fMRI. Neuroimage.

[R9] Berg D, Borghammer P, Fereshtehnejad SM, Heinzel S, Horsager J, Schaeffer E, Postuma RB (2021). Prodromal Parkinson disease subtypes - key to understanding heterogeneity. Nat Rev Neurol.

[R10] Burtscher J, Mallet RT, Burtscher M, Millet GP (2021). Hypoxia and brain aging: Neurodegeneration or neuroprotection?. Ageing Res Rev.

[R11] Chen J, Chen X, Cai H, Yang Y, Zhu Q, Sun D, Gao C (2025). The ubiquitination degradation of KLF15 mediated by WSB2 promotes lipogenesis and progression of hepatocellular carcinoma via inhibiting PDLIM2 expression. J Gastroenterol Hepatol.

[R12] Chu YP, Ho PC, Tsai KJ (2023). TTR (transthyretin) leads the autophagy disaster relief team against TARDBP/TDP-43 proteinopathy. Autophagy.

[R13] Coukos R, Krainc D (2024). Key genes and convergent pathogenic mechanisms in Parkinson disease. Nat Rev Neurosci.

[R14] Dash UC, Bhol NK, Swain SK, Samal RR, Nayak PK, Raina V, Panda SK, Kerry RG, Duttaroy AK, Jena AB (2024). Oxidative stress and inflammation in the pathogenesis of neurological disorders: Mechanisms and implications. Acta Pharm Sin B.

[R15] Dominguez Conde C (2022). Cross-tissue immune cell analysis reveals tissue-specific features in humans. Science.

[R16] Dosenbach NUF, Raichle ME, Gordon EM (2025). The brain’s action-mode network. Nat Rev Neurosci.

[R17] Edison P (2024). Astroglial activation: Current concepts and future directions. Alzheimers Dement.

[R18] Eira J, Magalhaes J, Macedo N, Pero ME, Misgeld T, Sousa MM, Bartolini F, Liz MA (2021). Transthyretin promotes axon growth via regulation of microtubule dynamics and tubulin acetylation. Front Cell Dev Biol.

[R19] Farokhian F, Beheshti I, Sone D, Matsuda H (2017). Comparing CAT12 and VBM8 for detecting brain morphological abnormalities in temporal lobe epilepsy. Front Neurol.

[R20] Foroutan M, Bhuva DD, Lyu R, Horan K, Cursons J, Davis MJ (2018). Single sample scoring of molecular phenotypes. BMC Bioinformatics.

[R21] Gao H, Nepovimova E, Heger Z, Valko M, Wu Q, Kuca K, Adam V (2023). Role of hypoxia in cellular senescence. Pharmacol Res.

[R22] Gaweda-Walerych K, Maruszak A, Safranow K, Bialecka M, Klodowska-Duda G, Czyzewski K, Slawek J, Rudzinska M, Styczynska M, Opala G, Drozdzik M, Canter JA, Barcikowska M, Zekanowski C (2008). Mitochondrial DNA haplogroups and subhaplogroups are associated with Parkinson’s disease risk in a Polish PD cohort. J Neural Transm (Vienna).

[R23] Gaweda-Walerych K, Safranow K, Maruszak A, Bialecka M, Klodowska-Duda G, Czyzewski K, Slawek J, Rudzinska M, Styczynska M, Opala G, Drozdzik M, Kurzawski M, Szczudlik A, Canter JA, Barcikowska M, Zekanowski C (2010). Mitochondrial transcription factor A variants and the risk of Parkinson’s disease. Neurosci Lett.

[R24] Giurgiu M, Reinhard J, Brauner B, Dunger-Kaltenbach I, Fobo G, Frishman G, Montrone C, Ruepp A (2019). CORUM: the comprehensive resource of mammalian protein complexes-2019. Nucleic Acids Res.

[R25] Gong J, Sun P, Li L, Zou Z, Wu Q, Sun L, Li H, Gu Z, Su L (2023). Heat stress suppresses MnSOD expression via p53-Sp1 interaction and induces oxidative stress damage in endothelial cells: Protective effects of MitoQ10 and Pifithrin-α. Heliyon.

[R26] Goudenège D, Bris C, Hoffmann V, Desquiret-Dumas V, Jardel C, Rucheton B, Bannwarth S, Paquis-Flucklinger V, Lebre AS, Colin E, Amati-Bonneau P, Bonneau D, Reynier P, Lenaers G, Procaccio V (2019). eKLIPse: a sensitive tool for the detection and quantification of mitochondrial DNA deletions from next-generation sequencing data. Genet Med.

[R27] Horsager J (2020). Brain-first versus body-first Parkinson’s disease: a multimodal imaging case-control study. Brain.

[R28] Hudson G, Nalls M, Evans JR, Breen DP, Winder-Rhodes S, Morrison KE, Morris HR, Williams-Gray CH, Barker RA, Singleton AB, Hardy J, Wood NE, Burn DJ, Chinnery PF (2013). Two-stage association study and meta-analysis of mitochondrial DNA variants in Parkinson disease. Neurology.

[R29] Ikeda A, Imai Y, Hattori N (2022). Neurodegeneration-associated mitochondrial proteins, CHCHD2 and CHCHD10-what distinguishes the two?. Front Cell Dev Biol.

[R30] Kim CY, Kim YC, Oh JH, Kim MH (2021). HOXA5 confers tamoxifen resistance via the PI3K/AKT signaling pathway in ER-positive breast cancer. J Cancer.

[R31] Kolde R, Laur S, Adler P, Vilo J (2012). Robust rank aggregation for gene list integration and meta-analysis. Bioinformatics.

[R32] Li L, Xu W, Zhang L (2021). KLF15 Regulates oxidative stress response in cardiomyocytes through NAD+. Metabolites.

[R33] Lim SY (2024). Uncovering the genetic basis of Parkinson’s disease globally: from discoveries to the clinic. Lancet Neurol.

[R34] Liu G (2023). Mitochondrial haplogroups and cognitive progression in Parkinson’s disease. Brain.

[R35] Mathys H (2023). Single-cell atlas reveals correlates of high cognitive function, dementia, and resilience to Alzheimer’s disease pathology. Cell.

[R36] Mohan A, Roberto AJ, Mohan A, Lorenzo A, Jones K, Carney MJ, Liogier-Weyback L, Hwang S, Lapidus KAB (2016). The significance of the default mode network (DMN) in neurological and neuropsychiatric disorders: a review. Yale J Biol Med.

[R37] Musgrove RE, Helwig M, Bae EJ, Aboutalebi H, Lee SJ, Ulusoy A, Di Monte DA (2019). Oxidative stress in vagal neurons promotes parkinsonian pathology and intercellular alpha-synuclein transfer. J Clin Invest.

[R38] N M P (2024). A multi-region single nucleus transcriptomic atlas of Parkinson’s disease. Sci Data.

[R39] Narendra DP, Youle RJ (2024). The role of PINK1-Parkin in mitochondrial quality control. Nat Cell Biol.

[R40] Nieto-Castanon A (2020). Handbook of functional connectivity magnetic resonance imaging methods in CONN.

[R41] Osorio D, Capasso A, Eckhardt SG, Giri U, Somma A, Pitts TM, Lieu CH, Messersmith WA, Bagby SM, Singh H, Das J, Sahni N, Yi SS, Kuijjer ML (2024). Population-level comparisons of gene regulatory networks modeled on high-throughput single-cell transcriptomics data. Nat Comput Sci.

[R42] Pinol RA, Mogul AS, Hadley CK, Saha A, Li C, Skop V, Province HS, Xiao C, Gavrilova O, Krashes MJ, Reitman ML (2021). Preoptic BRS3 neurons increase body temperature and heart rate via multiple pathways. Cell Metab.

[R43] Pinto-Costa R, Harbachova E, La Vitola P, Di Monte DA (2023). Overexpression-induced alpha-synuclein brain spreading. Neurotherapeutics.

[R44] Power JD, Mitra A, Laumann TO, Snyder AZ, Schlaggar BL, Petersen SE (2014). Methods to detect, characterize, and remove motion artifact in resting state fMRI. Neuroimage.

[R45] Priya Dharshini LC, Vishnupriya S, Sakthivel KM, Rasmi RR (2020). Oxidative stress responsive transcription factors in cellular signalling transduction mechanisms. Cell Signal.

[R46] Raichle ME, MacLeod AM, Snyder AZ, Powers WJ, Gusnard DA, Shulman GL (2001). A default mode of brain function. Proc Natl Acad Sci U S A.

[R47] Raman V, Martensen SA, Reisman D, Evron E, Odenwald WF, Jaffee E, Marks J, Sukumar S (2000). Compromised HOXA5 function can limit p53 expression in human breast tumours. Nature.

[R48] Rodriguez-Sabate C, Morales I, Sanchez A, Rodriguez M (2019). The functional interaction of the brain default network with motor networks is modified by aging. Behav Brain Res.

[R49] Schönherr S, Weissensteiner H, Kronenberg F, Forer L (2023). Haplogrep 3 - an interactive haplogroup classification and analysis platform. Nucleic Acids Res.

[R50] Searle SR, Speed FM, Milliken GA (2012). Population marginal means in the linear model: an alternative to least squares means. Am Stat.

[R51] Sena-Dos-Santos C, Moura DD, Epifane-de-Assuncao MC, Ribeiro-Dos-Santos A, Santos-Lobato BL (2024). Mitochondrial DNA variants, haplogroups and risk of Parkinson’s disease: A systematic review and meta-analysis. Parkinsonism Relat Disord.

[R52] Shetty NS, Gaonkar M, Patel N, Pampana A, Vekariya N, Li P, Arora G, Arora P (2024). Determinants of transthyretin levels and their association with adverse clinical outcomes among UK Biobank participants. Nat Commun.

[R53] Silva CS, Eira J, Ribeiro CA, Oliveira A, Sousa MM, Cardoso I, Liz MA (2017). Transthyretin neuroprotection in Alzheimer’s disease is dependent on proteolysis. Neurobiol Aging.

[R54] Sousa JC, Marques F, Dias-Ferreira E, Cerqueira JJ, Sousa N, Palha JA (2007). Transthyretin influences spatial reference memory. Neurobiol Learn Mem.

[R55] Squair JW, Gautier M, Kathe C, Anderson MA, James ND, Hutson TH, Hudelle R, Qaiser T, Matson KJE, Barraud Q, Levine AJ, La Manno G, Skinnider MA, Courtine G (2021). Confronting false discoveries in single-cell differential expression. Nat Commun.

[R56] Tansey MG, Wallings RL, Houser MC, Herrick MK, Keating CE, Joers V (2022). Inflammation and immune dysfunction in Parkinson disease. Nat Rev Immunol.

[R57] Wang Y, Xia Y, Kou L, Yin S, Chi X, Li J, Sun Y, Wu J, Zhou Q, Zou W, Jin Z, Huang J, Xiong N, Wang T (2023). Astrocyte-to-neuron reprogramming and crosstalk in the treatment of Parkinson’s disease. Neurobiol Dis.

[R58] Whitfield-Gabrieli S, Nieto-Castanon A (2012). Conn: a functional connectivity toolbox for correlated and anticorrelated brain networks. Brain Connect.

[R59] Wolf FA, Angerer P, Theis FJ (2018). SCANPY: large-scale single-cell gene expression data analysis. Genome Biol.

[R60] Wolock SL, Lopez R, Klein AM (2019). Scrublet: computational identification of cell doublets in single-cell transcriptomic data. Cell Syst.

[R61] Worsley KJ, Marrett S, Neelin P, Vandal AC, Friston KJ, Evans AC (1996). A unified statistical approach for determining significant signals in images of cerebral activation. Hum Brain Mapp.

[R62] Xiao B, Kuruvilla J, Tan EK (2022). Mitophagy and reactive oxygen species interplay in Parkinson’s disease. NPJ Parkinsons Dis.

[R63] Yamamoto J, Ikeda Y, Iguchi H, Fujino T, Tanaka T, Asaba H, Iwasaki S, Ioka RX, Kaneko IW, Magoori K, Takahashi S, Mori T, Sakaue H, Kodama T, Yanagisawa M, Yamamoto TT, Ito S, Sakai J (2004). A Krüppel-like factor KLF15 contributes fasting-induced transcriptional activation of mitochondrial acetyl-CoA synthetase gene AceCS2*. J Biol Chem.

[R64] Yang Y, Zhang Z (2024). alpha-Synuclein pathology from the body to the brain: so many seeds so close to the central soil. Neural Regen Res.

[R65] Ye H, Robak LA, Yu M, Cykowski M, Shulman JM (2023). Genetics and pathogenesis of Parkinson’s syndrome. Annu Rev Pathol.

[R66] Yeo BTT, Krienen FM, Sepulcre J, Sabuncu MR, Lashkari D, Hollinshead M, Roffman JL, Smoller JW, Zöllei L, Polimeni JR, Fischl B, Liu H, Buckner RL (2011). The organization of the human cerebral cortex estimated by intrinsic functional connectivity. J Neurophysiol.

[R67] Yi M, Nissley DV, McCormick F, Stephens RM (2020). ssGSEA score-based Ras dependency indexes derived from gene expression data reveal potential Ras addiction mechanisms with possible clinical implications. Sci Rep.

[R68] Zhang L, Zhao J, Zhou Q, Liu Z, Zhang Y, Cheng W, Gong W, Hu X, Lu W, Bullmore ET, Lo C-YZ, Feng J (2021). Sensory, somatomotor and internal mentation networks emerge dynamically in the resting brain with internal mentation predominating in older age. Neuroimage.

[R69] Zhang X, Qi Z, Yin H, Yang G (2019). Interaction between p53 and Ras signaling controls cisplatin resistance via HDAC4- and HIF-1alpha-mediated regulation of apoptosis and autophagy. Theranostics.

[R70] Zhao M, Wang S, Zuo A, Zhang J, Wen W, Jiang W, Chen H, Liang D, Sun J, Wang M (2021). HIF-1α/JMJD1A signaling regulates inflammation and oxidative stress following hyperglycemia and hypoxia-induced vascular cell injury. Cell Mol Biol Lett.

