# Supplementary material for: Hypoxia and TTR dysregulation in astrocytes from Parkinson’s disease with a specific mitochondrial haplogroup: A single-cell analysis
Source: Neural Regen Res. 2025 Sep 3;21(8):3807–14. doi: 10.4103/NRR.NRR-D-25-00107 (PMC13452667; doi:10.4103/NRR.NRR-D-25-00107)
Supplement: Supplementary file 1 [file NRR-21-3807_Suppl1.pdf]

Additional Table 3 Significant links of transcription factors affecting their target gene expression

tf	Target	Beta	*P*	FDR	Type	Celltype	Region
E2F4	CNTNAP3P2	2.75825	1.08E-07	0.049898495	Cluster	Ast CHI3L1	DMNX
E2F6	CNTNAP3P2	2.739916667	4.37E-08	0.046969109	Cluster	Ast CHI3L1	DMNX
ASCL1	CNTNAP3P2	2.6645	5.90E-08	0.049477641	Cluster	Ast CHI3L1	DMNX
IRF3	CNTNAP3P2	2.601166667	8.75E-08	0.049898495	Cluster	Ast CHI3L1	DMNX
PRDM4	CNTNAP3P2	-2.576166667	1.11E-07	0.049898495	Cluster	Ast CHI3L1	DMNX
PTF1A	CNTNAP3P2	2.40575	8.69E-08	0.049898495	Cluster	Ast CHI3L1	DMNX
PROX1	SPCS2	2.30375	5.92E-08	0.049477641	Cluster	Ast CHI3L1	DMNX
ATF7	CNTNAP3P2	-2.3	3.83E-08	0.046969109	Cluster	Ast CHI3L1	DMNX
GSC2	CNTNAP3P2	-2.288166667	3.51E-08	0.046969109	Cluster	Ast CHI3L1	DMNX
BSX	CNTNAP3P2	-2.2335	1.05E-07	0.049898495	Cluster	Ast CHI3L1	DMNX
BCL11A	TRDMT1	2.16425	9.54E-09	0.023905274	Cluster	Ast CHI3L1	DMNX
ZBTB49	CNTNAP3P2	-2.11025	6.34E-09	0.023905274	Cluster	Ast CHI3L1	DMNX
E2F3	CNTNAP3P2	1.889166667	8.51E-08	0.049898495	Cluster	Ast CHI3L1	DMNX
ELF3	CNTNAP3P2	1.578583333	6.71E-08	0.049898495	Cluster	Ast CHI3L1	DMNX
YY1	TRDMT1	1.5495	4.32E-08	0.046969109	Cluster	Ast CHI3L1	DMNX
ETV1	RHBDD3	-1.536666667	1.13E-07	0.049898495	Cluster	Ast CHI3L1	DMNX
ELK1	CYTH1	-1.197083333	7.93E-09	0.023905274	Cluster	Ast CHI3L1	DMNX
ZFX	TTR	4.3459375	2.23E-07	0.027473074	Cluster	Ast GRM3	DMNX
ZNF335	TTR	3.1493125	2.25E-07	0.027473074	Cluster	Ast GRM3	DMNX
ZNF320	TTR	3.1379375	2.25E-07	0.027473074	Cluster	Ast GRM3	DMNX
NFKB2	TTR	2.7265625	2.17E-07	0.027473074	Cluster	Ast GRM3	DMNX
ZBTB6	TTR	2.687375	2.12E-07	0.027473074	Cluster	Ast GRM3	DMNX
EGR4	TTR	2.6221875	2.63E-07	0.027473074	Cluster	Ast GRM3	DMNX
MBD2	TTR	2.6029375	3.55E-07	0.02877098	Cluster	Ast GRM3	DMNX
NFKB1	TTR	2.4459375	2.12E-07	0.027473074	Cluster	Ast GRM3	DMNX
HINFP	TTR	2.4036875	2.62E-07	0.027473074	Cluster	Ast GRM3	DMNX
GCM1	TTR	2.394375	2.50E-07	0.027473074	Cluster	Ast GRM3	DMNX
HOXA5	TTR	-2.3658125	4.70E-07	0.035120829	Cluster	Ast GRM3	DMNX
RELB	TTR	2.3263125	2.04E-07	0.027473074	Cluster	Ast GRM3	DMNX
IRF8	TTR	2.217	2.59E-07	0.027473074	Cluster	Ast GRM3	DMNX
STAT4	TTR	2.203125	2.65E-07	0.027473074	Cluster	Ast GRM3	DMNX
ZNF257	TTR	2.1836875	1.97E-07	0.027473074	Cluster	Ast GRM3	DMNX
STAT1	TTR	2.147	3.01E-07	0.027473074	Cluster	Ast GRM3	DMNX
MYCN	TTR	2.099375	2.23E-07	0.027473074	Cluster	Ast GRM3	DMNX
IRF4	TTR	2.0924375	2.57E-07	0.027473074	Cluster	Ast GRM3	DMNX
PROX1	TTR	-2.0854375	2.87E-07	0.027473074	Cluster	Ast GRM3	DMNX
BHLHE22	TTR	-2.081	1.44E-07	0.027473074	Cluster	Ast GRM3	DMNX
BHLHE23	TTR	-2.0725	1.48E-07	0.027473074	Cluster	Ast GRM3	DMNX
OLIG1	TTR	-2.0574375	1.85E-07	0.027473074	Cluster	Ast GRM3	DMNX
VAX2	TTR	-2.038375	5.85E-07	0.042281586	Cluster	Ast GRM3	DMNX
USF2	TTR	2.0309375	2.36E-07	0.027473074	Cluster	Ast GRM3	DMNX
EGR3	TTR	2.027125	2.31E-07	0.027473074	Cluster	Ast GRM3	DMNX
VAX1	TTR	-2.01175	6.06E-07	0.043052769	Cluster	Ast GRM3	DMNX
ONECUT2	TTR	-2.006625	4.04E-07	0.031276884	Cluster	Ast GRM3	DMNX
ZFP28	TTR	1.942875	2.39E-07	0.027473074	Cluster	Ast GRM3	DMNX
VDR	TTR	1.90975	4.95E-07	0.03655782	Cluster	Ast GRM3	DMNX
GATA5	TTR	-1.89	3.84E-07	0.030325291	Cluster	Ast GRM3	DMNX
MYC	TTR	1.8815	2.08E-07	0.027473074	Cluster	Ast GRM3	DMNX

MAFG	TTR	-1.8746875	1.09E-07	0.027473074	Cluster	Ast GRM3	DMNX
OLIG3	TTR	-1.8671875	2.17E-07	0.027473074	Cluster	Ast GRM3	DMNX
ZNF418	TTR	1.8553125	1.84E-07	0.027473074	Cluster	Ast GRM3	DMNX
ESX1	TTR	-1.830625	4.96E-07	0.03655782	Cluster	Ast GRM3	DMNX
IRX5	TTR	-1.8225	4.11E-07	0.031579836	Cluster	Ast GRM3	DMNX
EPAS1	TTR	-1.80675	3.56E-07	0.02877098	Cluster	Ast GRM3	DMNX
FOXF2	TTR	-1.801375	1.62E-07	0.027473074	Cluster	Ast GRM3	DMNX
IRX2	TTR	-1.80125	3.59E-07	0.02877098	Cluster	Ast GRM3	DMNX
HIC2	TTR	1.79125	1.54E-07	0.027473074	Cluster	Ast GRM3	DMNX
MEIS2	TTR	1.7840625	2.72E-07	0.027473074	Cluster	Ast GRM3	DMNX
BATF	TTR	-1.767875	1.35E-07	0.027473074	Cluster	Ast GRM3	DMNX
MEIS3	TTR	1.7524375	2.34E-07	0.027473074	Cluster	Ast GRM3	DMNX
TRAF4	TTR	-1.7494375	3.96E-07	0.030876081	Cluster	Ast GRM3	DMNX
NKX6-2	TTR	-1.7348125	4.58E-07	0.03466151	Cluster	Ast GRM3	DMNX
NEUROD2	TTR	-1.7286875	2.36E-07	0.027473074	Cluster	Ast GRM3	DMNX
PHOX2A	TTR	-1.72375	2.91E-07	0.027473074	Cluster	Ast GRM3	DMNX
TBP	TTR	-1.7185	1.18E-07	0.027473074	Cluster	Ast GRM3	DMNX
NEUROG1	TTR	-1.6950625	3.57E-07	0.02877098	Cluster	Ast GRM3	DMNX
EN2	TTR	-1.694625	3.01E-07	0.027473074	Cluster	Ast GRM3	DMNX
DRGX	TTR	-1.6945	3.42E-07	0.02877098	Cluster	Ast GRM3	DMNX
ONECUT3	TTR	-1.68675	2.52E-07	0.027473074	Cluster	Ast GRM3	DMNX
FOXP3	TTR	-1.6376875	2.29E-07	0.027473074	Cluster	Ast GRM3	DMNX
TCFL5	TTR	1.6375625	1.48E-07	0.027473074	Cluster	Ast GRM3	DMNX
PHOX2B	TTR	-1.636	2.96E-07	0.027473074	Cluster	Ast GRM3	DMNX
SOX7	TTR	-1.623	1.21E-07	0.027473074	Cluster	Ast GRM3	DMNX
FOS	TTR	1.6195	3.06E-07	0.027473074	Cluster	Ast GRM3	DMNX
FOXK2	TTR	-1.6178125	1.31E-07	0.027473074	Cluster	Ast GRM3	DMNX
NFAT5	TTR	1.613375	2.50E-07	0.027473074	Cluster	Ast GRM3	DMNX
FOXD1	TTR	-1.5885	1.62E-07	0.027473074	Cluster	Ast GRM3	DMNX
DMBX1	TTR	-1.580875	3.39E-07	0.02877098	Cluster	Ast GRM3	DMNX
HOXC9	TTR	-1.5748125	3.75E-07	0.029796679	Cluster	Ast GRM3	DMNX
RARB	TTR	-1.554	1.51E-07	0.027473074	Cluster	Ast GRM3	DMNX
GLI3	TTR	1.53025	2.03E-07	0.027473074	Cluster	Ast GRM3	DMNX
MECOM	TTR	-1.5140625	3.01E-07	0.027473074	Cluster	Ast GRM3	DMNX
PRRX2	TTR	-1.50575	3.96E-07	0.030876081	Cluster	Ast GRM3	DMNX
SOX8	TTR	-1.4635625	1.11E-07	0.027473074	Cluster	Ast GRM3	DMNX
PROP1	TTR	-1.438625	1.55E-07	0.027473074	Cluster	Ast GRM3	DMNX
DBP	TTR	-1.4194375	1.67E-07	0.027473074	Cluster	Ast GRM3	DMNX
CDX2	TTR	1.3961875	3.00E-07	0.027473074	Cluster	Ast GRM3	DMNX
RORC	TTR	1.3711875	1.99E-07	0.027473074	Cluster	Ast GRM3	DMNX
HES1	TTR	1.3663125	1.90E-07	0.027473074	Cluster	Ast GRM3	DMNX
CUX1	TTR	-1.366	2.43E-07	0.027473074	Cluster	Ast GRM3	DMNX
NRL	TTR	-1.3618125	1.43E-07	0.027473074	Cluster	Ast GRM3	DMNX
ZIM3	TTR	1.361	1.74E-07	0.027473074	Cluster	Ast GRM3	DMNX
CCR7	TTR	-1.3605625	3.55E-07	0.02877098	Cluster	Ast GRM3	DMNX
POU1F1	TTR	1.353625	3.52E-07	0.02877098	Cluster	Ast GRM3	DMNX
TWIST2	TTR	-1.349625	1.86E-07	0.027473074	Cluster	Ast GRM3	DMNX
MYBL2	TTR	-1.334	2.65E-07	0.027473074	Cluster	Ast GRM3	DMNX
ONECUT1	TTR	-1.3230625	1.23E-07	0.027473074	Cluster	Ast GRM3	DMNX
GATA6	TTR	-1.3141875	9.80E-08	0.027473074	Cluster	Ast GRM3	DMNX
POU4F1	TTR	-1.3105625	2.56E-07	0.027473074	Cluster	Ast GRM3	DMNX

ISX	TTR	-1.3055	2.84E-07	0.027473074	Cluster	Ast GRM3	DMNX
MLXIPL	TTR	-1.256875	3.49E-07	0.02877098	Cluster	Ast GRM3	DMNX
RELA	TTR	1.236	1.58E-07	0.027473074	Cluster	Ast GRM3	DMNX
PRRX1	TTR	-1.235125	1.96E-07	0.027473074	Cluster	Ast GRM3	DMNX
MEIS1	TTR	1.232	1.98E-07	0.027473074	Cluster	Ast GRM3	DMNX
HMX3	TTR	-1.2306875	2.31E-07	0.027473074	Cluster	Ast GRM3	DMNX
LHX9	TTR	-1.2225	2.84E-07	0.027473074	Cluster	Ast GRM3	DMNX
BATF3	TTR	1.2146875	2.62E-07	0.027473074	Cluster	Ast GRM3	DMNX
SRF	TTR	1.2089375	2.27E-07	0.027473074	Cluster	Ast GRM3	DMNX
FOXA3	TTR	-1.2076875	9.28E-08	0.027473074	Cluster	Ast GRM3	DMNX
MLXIP	TTR	-1.206625	1.63E-07	0.027473074	Cluster	Ast GRM3	DMNX
LBX2	TTR	-1.2056875	4.68E-07	0.035120829	Cluster	Ast GRM3	DMNX
SOX6	TTR	1.2001875	2.12E-07	0.027473074	Cluster	Ast GRM3	DMNX
TEAD2	TTR	1.1770625	1.83E-07	0.027473074	Cluster	Ast GRM3	DMNX
HSF4	TTR	-1.1763125	2.94E-07	0.027473074	Cluster	Ast GRM3	DMNX
POU6F2	TTR	-1.17	1.11E-07	0.027473074	Cluster	Ast GRM3	DMNX
DPRX	TTR	-1.161125	3.04E-07	0.027473074	Cluster	Ast GRM3	DMNX
SOX13	TTR	-1.1578125	2.28E-07	0.027473074	Cluster	Ast GRM3	DMNX
HIF1A	TTR	-1.1471875	1.71E-07	0.027473074	Cluster	Ast GRM3	DMNX
NFATC2	TTR	1.14125	1.98E-07	0.027473074	Cluster	Ast GRM3	DMNX
ID2	TTR	-1.1391875	1.98E-07	0.027473074	Cluster	Ast GRM3	DMNX
FOXM1	TTR	1.1209375	2.29E-07	0.027473074	Cluster	Ast GRM3	DMNX
HES5	TTR	1.1195625	1.95E-07	0.027473074	Cluster	Ast GRM3	DMNX
POU5F1B	TTR	-1.1061875	2.01E-07	0.027473074	Cluster	Ast GRM3	DMNX
NPAS2	TTR	-1.07525	1.32E-07	0.027473074	Cluster	Ast GRM3	DMNX
HOXD13	TTR	-1.06175	1.03E-07	0.027473074	Cluster	Ast GRM3	DMNX
NEUROG2	TTR	-1.0483125	1.18E-07	0.027473074	Cluster	Ast GRM3	DMNX
POU2F1	TTR	-1.039375	2.50E-07	0.027473074	Cluster	Ast GRM3	DMNX
CEBPG	TTR	1.0301875	2.81E-07	0.027473074	Cluster	Ast GRM3	DMNX
NKX2-3	TTR	1.010375	1.67E-07	0.027473074	Cluster	Ast GRM3	DMNX
POU3F4	TTR	0.9881875	2.03E-07	0.027473074	Cluster	Ast GRM3	DMNX
CEBPD	TTR	0.985875	2.02E-07	0.027473074	Cluster	Ast GRM3	DMNX
MEOX2	TTR	0.9856875	1.82E-07	0.027473074	Cluster	Ast GRM3	DMNX
TP53	TTR	-0.9651875	6.81E-07	0.047329928	Cluster	Ast GRM3	DMNX
HNF1B	TTR	-0.9593125	1.20E-07	0.027473074	Cluster	Ast GRM3	DMNX
HNF1A	TTR	-0.949	1.62E-07	0.027473074	Cluster	Ast GRM3	DMNX
TFCP2	TTR	-0.941625	2.49E-07	0.027473074	Cluster	Ast GRM3	DMNX
LHX6	TTR	-0.9365	1.53E-07	0.027473074	Cluster	Ast GRM3	DMNX
HOXC13	TTR	-0.9309375	1.31E-07	0.027473074	Cluster	Ast GRM3	DMNX
TAL1	TTR	-0.911375	1.27E-07	0.027473074	Cluster	Ast GRM3	DMNX
NKX3-1	TTR	-0.9106875	1.39E-07	0.027473074	Cluster	Ast GRM3	DMNX
HOXC12	TTR	-0.9098125	1.32E-07	0.027473074	Cluster	Ast GRM3	DMNX
HOXD12	TTR	-0.903375	1.23E-07	0.027473074	Cluster	Ast GRM3	DMNX
LHX2	TTR	0.901	1.84E-07	0.027473074	Cluster	Ast GRM3	DMNX
HOXB13	TTR	-0.882375	1.58E-07	0.027473074	Cluster	Ast GRM3	DMNX
ZNF582	TTR	-0.8788125	5.28E-07	0.038697972	Cluster	Ast GRM3	DMNX
FOXQ1	TTR	0.876875	2.99E-07	0.027473074	Cluster	Ast GRM3	DMNX
FOSB	TTR	0.8686875	1.85E-07	0.027473074	Cluster	Ast GRM3	DMNX
NFE2L2	TTR	-0.85225	2.77E-07	0.027473074	Cluster	Ast GRM3	DMNX
HES7	TTR	0.852	3.13E-07	0.027716151	Cluster	Ast GRM3	DMNX
POU5F1	TTR	-0.851	1.56E-07	0.027473074	Cluster	Ast GRM3	DMNX

CPEB1	TTR	-0.83225	2.02E-07	0.027473074	Cluster	Ast GRM3	DMNX
ZNF317	TTR	-0.8233125	3.39E-07	0.02877098	Cluster	Ast GRM3	DMNX
GATA3	TTR	-0.818375	2.87E-07	0.027473074	Cluster	Ast GRM3	DMNX
SOX15	TTR	0.7979375	2.22E-07	0.027473074	Cluster	Ast GRM3	DMNX
ZNF264	TTR	-0.782625	3.05E-07	0.027473074	Cluster	Ast GRM3	DMNX
CRX	TTR	-0.7619375	3.11E-07	0.027716151	Cluster	Ast GRM3	DMNX
FOXA2	TTR	-0.7479375	6.13E-07	0.043174134	Cluster	Ast GRM3	DMNX
DUX4	TTR	0.7335625	1.85E-07	0.027473074	Cluster	Ast GRM3	DMNX
CDX1	TTR	-0.73325	1.70E-07	0.027473074	Cluster	Ast GRM3	DMNX
LHX3	TTR	-0.728	2.11E-07	0.027473074	Cluster	Ast GRM3	DMNX
NR6A1	TTR	-0.726	2.86E-07	0.027473074	Cluster	Ast GRM3	DMNX
IRF9	TTR	0.72575	3.44E-07	0.02877098	Cluster	Ast GRM3	DMNX
ZNF382	TTR	-0.721125	6.07E-07	0.043052769	Cluster	Ast GRM3	DMNX
TCF21	TTR	0.718875	2.28E-07	0.027473074	Cluster	Ast GRM3	DMNX
NFIB	TTR	-0.710125	2.43E-07	0.027473074	Cluster	Ast GRM3	DMNX
ATF3	TTR	0.69075	5.40E-07	0.039271946	Cluster	Ast GRM3	DMNX
CREM	TTR	0.689375	4.30E-07	0.032770008	Cluster	Ast GRM3	DMNX
CEBPB	TTR	0.6730625	2.70E-07	0.027473074	Cluster	Ast GRM3	DMNX
TGIF2	TTR	0.6620625	1.80E-07	0.027473074	Cluster	Ast GRM3	DMNX
EN1	TTR	-0.645	3.20E-07	0.028057252	Cluster	Ast GRM3	DMNX
BARHL2	TTR	0.63275	3.29E-07	0.028687906	Cluster	Ast GRM3	DMNX
ALX3	TTR	0.5965625	2.70E-07	0.027473074	Cluster	Ast GRM3	DMNX
HOXC11	TTR	0.588875	2.36E-07	0.027473074	Cluster	Ast GRM3	DMNX
EMX1	TTR	0.5601875	2.25E-07	0.027473074	Cluster	Ast GRM3	DMNX
CREB3	TTR	0.5265	6.24E-07	0.04370633	Cluster	Ast GRM3	DMNX
SOX5	TTR	-0.52475	6.01E-07	0.043052769	Cluster	Ast GRM3	DMNX
GLIS1	NAT10	-2.381986111	4.63E-07	0.047622249	Cluster	Ast DPP10	GPI
SOX11	NAT10	-2.245902778	3.18E-07	0.037815843	Cluster	Ast DPP10	GPI
ZNF250	NAT10	-2.0535	2.80E-07	0.037795796	Cluster	Ast DPP10	GPI
HOMEZ	NAT10	-2.018555556	3.05E-07	0.037795796	Cluster	Ast DPP10	GPI
HOXA11	NAT10	-1.932486111	3.05E-07	0.037795796	Cluster	Ast DPP10	GPI
VSX2	NAT10	-1.897069444	3.06E-07	0.037795796	Cluster	Ast DPP10	GPI
HOXB2	NAT10	-1.842375	2.97E-07	0.037795796	Cluster	Ast DPP10	GPI
EVX1	NAT10	-1.840652778	2.96E-07	0.037795796	Cluster	Ast DPP10	GPI
DLX1	NAT10	-1.832208333	2.03E-07	0.030503498	Cluster	Ast DPP10	GPI
DLX5	NAT10	-1.80025	1.70E-07	0.030503498	Cluster	Ast DPP10	GPI
DLX6	NAT10	-1.791097222	2.01E-07	0.030503498	Cluster	Ast DPP10	GPI
PAX4	NAT10	-1.788430556	1.86E-07	0.030503498	Cluster	Ast DPP10	GPI
HOXA1	NAT10	-1.787458333	1.69E-07	0.030503498	Cluster	Ast DPP10	GPI
LHX4	NAT10	-1.779833333	1.66E-07	0.030503498	Cluster	Ast DPP10	GPI
RAX2	NAT10	-1.774986111	3.06E-07	0.037795796	Cluster	Ast DPP10	GPI
CUX2	NAT10	-1.772736111	3.14E-07	0.037815843	Cluster	Ast DPP10	GPI
VAX1	NAT10	-1.763	4.51E-07	0.046832286	Cluster	Ast DPP10	GPI
HOXA2	NAT10	-1.757402778	2.11E-07	0.031314055	Cluster	Ast DPP10	GPI
ZNF502	NAT10	-1.751819444	3.75E-07	0.04198765	Cluster	Ast DPP10	GPI
MIXL1	NAT10	-1.751805556	1.45E-07	0.030503498	Cluster	Ast DPP10	GPI
PRDM4	NAT10	-1.728611111	2.55E-07	0.035486463	Cluster	Ast DPP10	GPI
HMBOX1	NAT10	-1.712486111	1.33E-07	0.030503498	Cluster	Ast DPP10	GPI
MEOX1	NAT10	-1.694	1.87E-07	0.030503498	Cluster	Ast DPP10	GPI
HOXB3	NAT10	-1.692652778	2.34E-07	0.03290604	Cluster	Ast DPP10	GPI
VENTX	NAT10	-1.659513889	2.90E-07	0.037795796	Cluster	Ast DPP10	GPI

GSC2	NAT10	-1.657111111	1.51E-07	0.030503498	Cluster	Ast DPP10	GPI
GBX1	NAT10	-1.643458333	1.96E-07	0.030503498	Cluster	Ast DPP10	GPI
LHX8	NAT10	-1.630611111	1.42E-07	0.030503498	Cluster	Ast DPP10	GPI
ONECUT2	NAT10	-1.621736111	1.41E-07	0.030503498	Cluster	Ast DPP10	GPI
LMX1A	NAT10	-1.60675	1.32E-07	0.030503498	Cluster	Ast DPP10	GPI
ESX1	NAT10	-1.606736111	1.58E-07	0.030503498	Cluster	Ast DPP10	GPI
PAX3	NAT10	-1.582333333	1.08E-07	0.030503498	Cluster	Ast DPP10	GPI
RAX	NAT10	-1.580694444	2.02E-07	0.030503498	Cluster	Ast DPP10	GPI
BSX	NAT10	-1.575527778	1.90E-07	0.030503498	Cluster	Ast DPP10	GPI
GATA5	NAT10	-1.569638889	9.49E-08	0.030503498	Cluster	Ast DPP10	GPI
GSC	NAT10	-1.547291667	1.22E-07	0.030503498	Cluster	Ast DPP10	GPI
ZNF136	NAT10	-1.546819444	3.51E-07	0.040280442	Cluster	Ast DPP10	GPI
PAX7	NAT10	-1.52925	1.23E-07	0.030503498	Cluster	Ast DPP10	GPI
PROX1	NAT10	-1.510888889	2.33E-07	0.03290604	Cluster	Ast DPP10	GPI
SOX1	NAT10	-1.504444444	7.36E-08	0.030503498	Cluster	Ast DPP10	GPI
ZNF260	NAT10	-1.496180556	2.62E-07	0.035937777	Cluster	Ast DPP10	GPI
IRX5	NAT10	-1.490388889	1.94E-07	0.030503498	Cluster	Ast DPP10	GPI
MNX1	NAT10	-1.480902778	1.67E-07	0.030503498	Cluster	Ast DPP10	GPI
DUXA	NAT10	-1.470083333	8.46E-08	0.030503498	Cluster	Ast DPP10	GPI
NKX6-1	NAT10	-1.463777778	1.13E-07	0.030503498	Cluster	Ast DPP10	GPI
IRX2	NAT10	-1.460555556	1.77E-07	0.030503498	Cluster	Ast DPP10	GPI
TRAF4	NAT10	-1.448958333	1.81E-07	0.030503498	Cluster	Ast DPP10	GPI
MLX	NAT10	-1.403402778	9.02E-08	0.030503498	Cluster	Ast DPP10	GPI
TEF	NAT10	-1.403333333	9.52E-08	0.030503498	Cluster	Ast DPP10	GPI
DMRT3	NAT10	-1.386569444	1.25E-07	0.030503498	Cluster	Ast DPP10	GPI
ZBTB49	NAT10	-1.381444444	3.42E-07	0.039846404	Cluster	Ast DPP10	GPI
LMX1B	NAT10	-1.380319444	8.12E-08	0.030503498	Cluster	Ast DPP10	GPI
OVOL1	NAT10	-1.367027778	1.07E-07	0.030503498	Cluster	Ast DPP10	GPI
ARID5A	NAT10	-1.353847222	6.30E-08	0.030503498	Cluster	Ast DPP10	GPI
DRGX	NAT10	-1.333569444	1.25E-07	0.030503498	Cluster	Ast DPP10	GPI
EN2	NAT10	-1.327472222	7.30E-08	0.030503498	Cluster	Ast DPP10	GPI
PHOX2A	NAT10	-1.312347222	8.71E-08	0.030503498	Cluster	Ast DPP10	GPI
ISL2	NAT10	-1.300291667	1.33E-07	0.030503498	Cluster	Ast DPP10	GPI
DMBX1	NAT10	-1.277986111	1.58E-07	0.030503498	Cluster	Ast DPP10	GPI
ATF1	NAT10	-1.269666667	1.16E-07	0.030503498	Cluster	Ast DPP10	GPI
ARID3B	NAT10	-1.267430556	8.97E-08	0.030503498	Cluster	Ast DPP10	GPI
HOXC9	NAT10	-1.254888889	9.37E-08	0.030503498	Cluster	Ast DPP10	GPI
VSX1	NAT10	-1.252222222	1.90E-07	0.030503498	Cluster	Ast DPP10	GPI
HOXA9	NAT10	-1.240333333	1.06E-07	0.030503498	Cluster	Ast DPP10	GPI
CREB3L2	NAT10	-1.237930556	3.04E-07	0.037795796	Cluster	Ast DPP10	GPI
PHOX2B	NAT10	-1.236625	8.06E-08	0.030503498	Cluster	Ast DPP10	GPI
FOXP3	NAT10	-1.216972222	4.16E-08	0.030503498	Cluster	Ast DPP10	GPI
GMEB2	NAT10	-1.214833333	1.54E-07	0.030503498	Cluster	Ast DPP10	GPI
PITX3	NAT10	-1.213291667	4.20E-07	0.04452543	Cluster	Ast DPP10	GPI
ONECUT3	NAT10	-1.210472222	7.95E-08	0.030503498	Cluster	Ast DPP10	GPI
HOXB5	NAT10	-1.19525	9.72E-08	0.030503498	Cluster	Ast DPP10	GPI
E2F8	NAT10	-1.187444444	1.71E-07	0.030503498	Cluster	Ast DPP10	GPI
ETV3	NAT10	-1.178861111	3.85E-07	0.04198765	Cluster	Ast DPP10	GPI
HLF	NAT10	-1.175708333	4.32E-08	0.030503498	Cluster	Ast DPP10	GPI
PKNOX1	NAT10	-1.157861111	1.83E-07	0.030503498	Cluster	Ast DPP10	GPI
SIX3	NAT10	-1.153527778	1.00E-07	0.030503498	Cluster	Ast DPP10	GPI

ELK3	NAT10	-1.140111111	4.38E-07	0.045926698	Cluster	Ast DPP10	GPI
CCR7	NAT10	-1.095888889	1.02E-07	0.030503498	Cluster	Ast DPP10	GPI
HOXD9	NAT10	-1.092375	5.56E-08	0.030503498	Cluster	Ast DPP10	GPI
SOX18	NAT10	-1.060055556	1.14E-07	0.030503498	Cluster	Ast DPP10	GPI
GLIS3	NAT10	-1.057388889	1.72E-07	0.030503498	Cluster	Ast DPP10	GPI
MECOM	NAT10	-1.055333333	3.20E-07	0.037815843	Cluster	Ast DPP10	GPI
ERF	NAT10	-1.047111111	1.58E-07	0.030503498	Cluster	Ast DPP10	GPI
FOXF2	NAT10	-1.028861111	1.07E-07	0.030503498	Cluster	Ast DPP10	GPI
DLX3	NAT10	-1.015875	1.15E-07	0.030503498	Cluster	Ast DPP10	GPI
FOXD1	NAT10	-1.000694444	9.09E-08	0.030503498	Cluster	Ast DPP10	GPI
ZSCAN4	NAT10	-0.995361111	3.82E-07	0.04198765	Cluster	Ast DPP10	GPI
NKX3-2	NAT10	-0.993944444	6.70E-08	0.030503498	Cluster	Ast DPP10	GPI
BATF	NAT10	-0.983986111	1.64E-08	0.030503498	Cluster	Ast DPP10	GPI
ZNF680	NAT10	-0.979513889	3.18E-07	0.037815843	Cluster	Ast DPP10	GPI
ISX	NAT10	-0.97275	3.44E-07	0.039846404	Cluster	Ast DPP10	GPI
FOXK2	NAT10	-0.954138889	4.83E-08	0.030503498	Cluster	Ast DPP10	GPI
DBP	NAT10	-0.903652778	8.59E-08	0.030503498	Cluster	Ast DPP10	GPI
RARB	NAT10	-0.899708333	1.62E-07	0.030503498	Cluster	Ast DPP10	GPI
KLF13	NAT10	-0.897652778	1.83E-07	0.030503498	Cluster	Ast DPP10	GPI
HMX3	NAT10	-0.895763889	1.86E-07	0.030503498	Cluster	Ast DPP10	GPI
SCRT2	NAT10	-0.893208333	4.07E-07	0.043492013	Cluster	Ast DPP10	GPI
NRL	NAT10	-0.887486111	9.88E-08	0.030503498	Cluster	Ast DPP10	GPI
HMX2	NAT10	-0.844486111	1.77E-07	0.030503498	Cluster	Ast DPP10	GPI
MYBL1	NAT10	-0.842430556	3.92E-07	0.042365208	Cluster	Ast DPP10	GPI
TFEC	NAT10	-0.836444444	3.58E-07	0.040576024	Cluster	Ast DPP10	GPI
HOXA10	NAT10	-0.832583333	1.23E-07	0.030503498	Cluster	Ast DPP10	GPI
PRRX1	NAT10	-0.831319444	2.19E-07	0.032037063	Cluster	Ast DPP10	GPI
GBX2	NAT10	-0.798305556	1.36E-07	0.030503498	Cluster	Ast DPP10	GPI
HOXD11	NAT10	-0.786194444	4.77E-07	0.048645226	Cluster	Ast DPP10	GPI
FOXA3	NAT10	-0.753152778	1.17E-07	0.030503498	Cluster	Ast DPP10	GPI
ELF4	NAT10	-0.739055556	2.28E-07	0.0328706	Cluster	Ast DPP10	GPI
PRRX1	TMEM67	-0.632597222	1.89E-07	0.030503498	Cluster	Ast DPP10	GPI
ARX	TMEM67	-0.561888889	3.80E-07	0.04198765	Cluster	Ast DPP10	GPI
FLI1	CCNE1	3.010666667	1.06E-07	0.032816554	Cluster	Ast CHI3L1	PMC
CREB3L1	NPM2	-2.78975	3.21E-08	0.0232275	Cluster	Ast CHI3L1	PMC
E2F3	LINC00888	-2.561083333	1.90E-07	0.044297527	Cluster	Ast CHI3L1	PMC
POU1F1	EXOSC4	2.29375	1.07E-07	0.032816554	Cluster	Ast CHI3L1	PMC
TFEB	NPM2	-2.23625	1.49E-07	0.040144265	Cluster	Ast CHI3L1	PMC
NEUROG2	LINC00836	-2.13975	1.75E-07	0.044010477	Cluster	Ast CHI3L1	PMC
BHLHE41	NPM2	-2.0845	8.76E-08	0.031700142	Cluster	Ast CHI3L1	PMC
HES5	EXOSC4	1.95925	1.04E-07	0.032816554	Cluster	Ast CHI3L1	PMC
NFYA	EXOSC4	1.9325	8.81E-08	0.031700142	Cluster	Ast CHI3L1	PMC
POU2F3	EXOSC4	1.92875	6.07E-08	0.028543248	Cluster	Ast CHI3L1	PMC
POU3F1	EXOSC4	1.90575	6.37E-08	0.028543248	Cluster	Ast CHI3L1	PMC
TP53	GAGE10	1.88575	8.25E-08	0.031700142	Cluster	Ast CHI3L1	PMC
POU2F1	EXOSC4	1.84225	1.17E-07	0.034712393	Cluster	Ast CHI3L1	PMC
PDX1	EXOSC4	1.83875	3.61E-08	0.02388914	Cluster	Ast CHI3L1	PMC
NFATC2	EXOSC4	1.80525	4.94E-08	0.028543248	Cluster	Ast CHI3L1	PMC
NKX2-3	EXOSC4	1.78975	1.07E-07	0.032816554	Cluster	Ast CHI3L1	PMC
POU3F2	EXOSC4	1.7755	3.10E-08	0.0232275	Cluster	Ast CHI3L1	PMC
HOXA13	TMEM225B	1.76925	1.82E-07	0.044297527	Cluster	Ast CHI3L1	PMC

HES1	GAGE10	1.76375	1.59E-08	0.019047273	Cluster	Ast CHI3L1	PMC
ZNF260	CCNE1	-1.762083333	5.77E-08	0.028543248	Cluster	Ast CHI3L1	PMC
PDX1	TMEM225B	1.757	1.87E-07	0.044297527	Cluster	Ast CHI3L1	PMC
NR6A1	CHST14	1.7275	8.97E-08	0.031700142	Cluster	Ast CHI3L1	PMC
BHLHE41	EXOSC4	1.724	6.18E-08	0.028543248	Cluster	Ast CHI3L1	PMC
SOX14	EXOSC4	1.69725	1.48E-07	0.040144265	Cluster	Ast CHI3L1	PMC
POU5F1B	EXOSC4	1.66575	1.43E-08	0.019047273	Cluster	Ast CHI3L1	PMC
HES5	NPM2	-1.66325	1.15E-08	0.017736971	Cluster	Ast CHI3L1	PMC
CRX	TMEM225B	1.65775	5.71E-09	0.017736971	Cluster	Ast CHI3L1	PMC
GATA3	TMEM225B	1.643	1.09E-08	0.017736971	Cluster	Ast CHI3L1	PMC
BHLHE41	TMEM225B	1.631	2.13E-07	0.047661914	Cluster	Ast CHI3L1	PMC
TCF7L2	EXOSC4	1.622	2.09E-08	0.022476516	Cluster	Ast CHI3L1	PMC
SOX5	EXOSC4	1.6125	1.94E-09	0.017736971	Cluster	Ast CHI3L1	PMC
GATA6	NPM2	-1.556	2.94E-08	0.0232275	Cluster	Ast CHI3L1	PMC
POU3F4	EXOSC4	1.55125	2.63E-08	0.0232275	Cluster	Ast CHI3L1	PMC
TFCP2	TMEM225B	1.54025	1.19E-07	0.034712393	Cluster	Ast CHI3L1	PMC
FOXA3	NPM2	-1.5315	5.63E-08	0.028543248	Cluster	Ast CHI3L1	PMC
NR6A1	TMEM225B	1.51825	3.78E-08	0.02388914	Cluster	Ast CHI3L1	PMC
NANOG	TMEM225B	1.496	9.14E-08	0.031700142	Cluster	Ast CHI3L1	PMC
ATF6	GCHFR	1.47025	4.08E-09	0.017736971	Cluster	Ast CHI3L1	PMC
TCF7L1	SLC25A41	1.455	1.76E-07	0.044010477	Cluster	Ast CHI3L1	PMC
CEBPB	SLC25A41	1.3775	1.08E-08	0.017736971	Cluster	Ast CHI3L1	PMC
HMX2	NPM2	-1.359	2.25E-07	0.049365443	Cluster	Ast CHI3L1	PMC
POU5F1	SLC25A41	1.3445	7.79E-08	0.031700142	Cluster	Ast CHI3L1	PMC
CDX1	SLC25A41	1.31475	1.24E-07	0.035219597	Cluster	Ast CHI3L1	PMC
NFATC3	NPM2	1.153	6.23E-08	0.028543248	Cluster	Ast CHI3L1	PMC
GBX2	NPM2	1.118	7.17E-09	0.017736971	Cluster	Ast CHI3L1	PMC
THRB	NPM2	-1.09125	8.84E-08	0.031700142	Cluster	Ast CHI3L1	PMC
HOXB13	SLC25A41	-1.0625	1.97E-07	0.044978377	Cluster	Ast CHI3L1	PMC
MSX1	ELMO3	0.884583333	3.24E-08	0.0232275	Cluster	Ast CHI3L1	PMC
BARX1	LCA5L	-0.7975	1.61E-07	0.042167994	Cluster	Ast CHI3L1	PMC
